# SEPALLATA3: the 'glue' for MADS box transcription factor complex formation

**DOI:** 10.1186/gb-2009-10-2-r24

**Published:** 2009-02-25

**Authors:** Richard GH Immink, Isabella AN Tonaco, Stefan de Folter, Anna Shchennikova, Aalt DJ van Dijk, Jacqueline Busscher-Lange, Jan W Borst, Gerco C Angenent

**Affiliations:** 1 Plant Research International, Bioscience, Droevendaalsesteeg 1, Wageningen, the Netherlands; 2 Wageningen University, Microspectroscopy Centre, Department of Biochemistry, Dreijenlaan 3, Wageningen, the Netherlands; 3 Current address: National Laboratory of Genomics for Biodiversity (Langebio), Centro de Investigación y de Estudios Avanzados del Instituto Politécnico Nacional (CINVESTAV-IPN), Campus Guanajuato, CP 36821 Irapuato, Guanajuato, Mexico; 4 Current address: Center 'Bioengineering' RAS, prospect 60-letia Oktyabrya, 7, korp. 1, 117321 Moscow, Russia

## Abstract

**Background:**

Plant MADS box proteins play important roles in a plethora of developmental processes. In order to regulate specific sets of target genes, MADS box proteins dimerize and are thought to assemble into multimeric complexes. In this study a large-scale yeast three-hybrid screen is utilized to provide insight into the higher-order complex formation capacity of the *Arabidopsis *MADS box family. SEPALLATA3 (SEP3) has been shown to mediate complex formation and, therefore, special attention is paid to this factor in this study.

**Results:**

In total, 106 multimeric complexes were identified; in more than half of these at least one SEP protein was present. Besides the known complexes involved in determining floral organ identity, various complexes consisting of combinations of proteins known to play a role in floral organ identity specification, and flowering time determination were discovered. The capacity to form this latter type of complex suggests that homeotic factors play essential roles in down-regulation of the MADS box genes involved in floral timing in the flower via negative auto-regulatory loops. Furthermore, various novel complexes were identified that may be important for the direct regulation of the floral transition process. A subsequent detailed analysis of the APETALA3, PISTILLATA, and SEP3 proteins in living plant cells suggests the formation of a multimeric complex *in vivo*.

**Conclusions:**

Overall, these results provide strong indications that higher-order complex formation is a general and essential molecular mechanism for plant MADS box protein functioning and attribute a pivotal role to the SEP3 'glue' protein in mediating multimerization.

## Abstract 2

A yeast 3-hybrid screen in Arabidopsis reveals MADS box protein complexes: SEP3 is shown to mediate complex formation and floral timing.

## Background

Since the isolation of the first plant MADS box transcription factor gene, substantial knowledge has been gained about the biological functions of these developmental regulators in various plant species. A thorough analysis of the complete genome sequence from the model species *Arabidopsis thaliana *revealed the presence of 107 different members belonging to this transcription factor family, with known or predicted functions in floral induction, plant architecture, female gametophyte development, fruit formation, fruit ripening, pod shattering, nitrate signaling and floral organ development [1-3]. Already in the early 1990s, genetic studies using floral organ mutants in *Arabidopsis *and *Antirrhinum majus*, representing mutations in mainly MADS box transcription factor genes, led to the establishment of the robust 'ABC model' for floral organ formation [4]. According to this original model, organ identities are determined by combinations of three functions, in which the A-function is essential for the specification of sepal identity, A- and B-functions for petals, B- and C-functions determine stamen identity, and the C-function on its own is responsible for carpel formation. In *Arabidopsis *the A-function is defined by *APETALA1 *(*AP1*) and *APETALA2 *(*AP2*), the B-function by *APETALA3 *(*AP3*) and *PISTILLATA *(*PI*), and the C-function by *AGAMOUS *(*AG*), from which only the *AP2 *gene does not belong to the MADS box family.

Although the original 'ABC model' describes well the homeotic mutations in the various floral mutants, the lack of floral organ formation outside the flower when B- and/or C-function MADS box genes were ectopically expressed indicated that more factors are required for the floral organ identity functions [5,6]. In *Arabidopsis*, the *SEPALLATA *(*SEP*) MADS box genes appeared to be the missing co-factors and this new class of floral organ identity genes was termed E-function genes [7]. In line with the refined and extended 'ABC model', combinatorial over-expression of A-, B- and E-function genes results in conversion of leaves into petals, whereas constitutive expression of B-, C- and E-function genes gives rise to the formation of stamens instead of leaves [8-10]. Like for many MADS box genes, functional redundancy exists for the E-function genes, and only in the *sep1 sep2 sep3 *triple mutant were clear phenotypical alterations observed, namely the conversion of the second and third whorl organs into sepals and the development of a new inflorescence from the central region of the floral meristem [7]. Mutation of the fourth *Arabidopsis SEP *gene (*SEP4*) in a *sep1 sep2 sep3 *background resulted in the production of leaves only [11] and reveals an important function for *SEP4 *in sepal development. In addition, these latter observations give supporting evidence for Goethe's so-called 'big metamorphose', which proposes that a genetic program for the development of leaves is the basis for the formation of the flower, implying that floral organs can be regarded as modified leaves [12]. More detailed analyses of double and triple *sep4*, *cauliflower *(*cal*), and* ap1 *mutants and genetic titration experiments for the *sep *mutations demonstrated that *SEP4 *also has a role in establishing floral meristem identity and petal, stamen and carpel development [11]. Furthermore, the genetic titration experiments for the *sep *mutations described by Ditta and colleagues [11] showed dosage effects and redundancy for the *SEP *genes. Similar conclusions were drawn in relation to ovule development, in which the *SEP *genes act in a dose-dependent manner together with the C-function gene *AG *and the D-function genes *SEEDSTICK *(*STK*), *SHATTERPROOF1 *(*SHP1*) and *SHATTERPROOF2 *(*SHP2*) [13].

In conclusion, all these genetic data point towards a central role for the *SEP *genes in floral meristem and floral organ development. The importance of this class of genes for floral development has been put forward from an evolutionary point of view as well. Based on detailed phylogenetic studies and the fact that *SEP *like genes have been isolated from angiosperms only, Zahn and colleagues [14] suggested that the *SEP *genes might be the basis for the origin of flowers.

An intriguing question arising from the ABC model is how all these different MADS box transcription factors co-operate together at the molecular level. Part of this question could be answered based on *in vitro *biochemical assays [15] and yeast two-, three- and four-hybrid experiments that were performed over the past decade (among others [8,16,17]). The yeast experiments revealed binary interactions between specific A-, B-, C-, D-, and E-function MADS box proteins and, furthermore, they suggest the assembly into higher-order complexes consisting of 'ABC'-function MADS domain proteins and dimers. These results support the notion that MADS box proteins are active in a combinatorial manner and, accordingly, the 'Quartet model' has been proposed for MADS box transcription factor functioning [18]. In this model, a pivotal role has been attributed to the SEP proteins (E-function), which are present in almost all known higher-order complexes and, thus, can be regarded as the 'glue' proteins of floral organ development. Similar higher-order complexes have been identified for MADS box proteins of other species, such as *Antirrhinum *[17], chrysanthemum [19], petunia [20-23] and tomato [24], demonstrating that these types of interactions are conserved among angiosperm species. Furthermore, it has been shown recently that the SEP3 protein on its own is able to form homotetramers *in vitro *[25]. Based on all these findings, it is acceptable to use the 'Quartet model' as the working model for MADS box transcription factor functioning, although hardly any evidence for direct physical higher-order complex formation between MADS proteins in plant cells has been found. Recently, it has been shown that the transient interaction between the petunia MADS box proteins FLORAL BINDING PROTEIN11 (FBP11) and FBP24 in protoplasts can be stabilized by adding the FBP2 protein, suggesting that a multimeric protein complex is formed in living plant cells [23]. Furthermore, gel filtration experiments with native protein extracts revealed that the FLOWERING LOCUS C (FLC) MADS box transcription factor is present in high molecular weight complexes [26]. In conclusion, MADS box proteins are able to multimerize in plant cells and are present in large complexes *in vivo*; however, the exact composition and stoichiometry of these complexes remains unknown.

In this study a large-scale yeast three-hybrid screen was performed to unravel the capacity and selectivity of higher-order complex formation for *Arabidopsis *MADS box transcription factors, with a special focus on the SEP proteins. In total, 106 ternary interactions were scored and in 78 cases at least one SEP protein appeared to be involved. The obtained results illustrate that higher-order complex formation is common among MADS proteins, and that this mechanism is employed by all subfamilies of the MADS box family. Based on available expression data for the MADS box genes that code for the interacting proteins, previous mutant analyses, and interaction studies in living plant cells, biological functions could be proposed for particular SEP3 complexes.

## Results

### Large scale yeast three-hybrid analysis

After the discovery that *A. majus *MADS box proteins are able to form multimeric complexes in yeast [17], a small number of additional ternary and quaternary complexes has been identified for MADS box proteins from various species. Currently, approximately 20 potential higher-order complexes involving *Arabidopsis *MADS box proteins have been reported [8,13,20,27] (Table S1 in Additional data file 1). Remarkably, the vast majority of these complexes contains the SEP3 protein, which suggests that proteins of this sub-clade are important mediators of higher-order complex formation.

To get a better understanding about the capacity and specificity of complex formation for *Arabidopsis *MADS box proteins in general, and for the SEP3 protein in particular, a large scale yeast three-hybrid screening was performed. For this purpose all MADS box protein dimers that were identified in the comprehensive yeast two-hybrid screening [16] were reconstituted in yeast strain PJ69-4 mating type A (Table S2 in Additional data file 1) by expressing one of the two dimerization partners as a fusion with the activation domain (AD) of the yeast GAL4 transcription factor, while the other protein was fused to a nuclear localization signal only [28]. Subsequently, these yeast clones were screened against the available collection of single MADS box proteins fused to the GAL4 binding domain (BD) in yeast strain PJ69-4 mating type Alpha [16].

In total, 27,400 combinations (274 dimers × 100 single proteins) were tested for ternary complex formation and this screen yielded 47 positives (Table S3 in Additional data file 1). The results reveal a preference for ternary complex formation with proteins of the same sub-class of MADS box proteins; in general, type II proteins interact with other type II proteins and the same holds for members of the type I sub-class. Besides the 47 higher-order complexes that were identified in this screen, nine additional dimers were found that were missed in the large-scale yeast two-hybrid screening performed by De Folter and colleagues [16] (Table S4 in Additional data file 1). Most likely, this difference is caused by the more mild selection criteria used for the yeast three-hybrid experiments. Although, many new triple combinations were found, the total number of ternary interactions was much lower than expected and, to our surprise, none of the known complexes was identified. The latter discrepancy could be explained to a large extent by technical limitations of the system: many combinations could not be tested for ternary complex formation, because the two proteins that were fused to GAL4-AD and -BD were already able to form a dimer that activated the yeast reporter genes even without the incorporation of the third protein in the complex. For instance, we could not observe the interaction between SEP3, STK (dimer 257 in Table S2 in Additional data file 1) and AG [13], because GAL4-AD-SEP3 and GAL4-BD-AG are able to dimerize and activate the yeast reporter [16]. Furthermore, the presence of an intrinsic transcriptional AD in about 20% of the *Arabidopsis *MADS box proteins [16], including the SEP1 and SEP3 proteins [10], limited drastically the number of combinations that could be tested for ternary interactions due to auto-activation of the yeast reporters.

### SEP3 ternary complex formation

One of the main goals of the large-scale yeast three-hybrid screening was to obtain a comprehensive picture of the potential of SEP proteins to mediate higher-order complex formation. However, this objective was hampered by the large number of dimers formed by these proteins and auto-activation of the yeast reporters by the SEP proteins. To overcome the latter problem we mapped the auto-activation domain in the SEP3 protein in order to remove this domain from the protein. This SEP member was chosen because genetic studies [7,11], transactivation assays [10], and yeast two-hybrid experiments [16] have revealed that SEP3 is the most 'active' member of the SEP clade. To predict the presence of potential transcriptional activation domains, a search for motifs was performed with the software program DILIMOT on the full-length sequences of all MADS box proteins that gave auto-activation in yeast [16]. In this screen, a total of ten motifs was found, including the ones that were identified for the AP1 protein previously [29], and almost all appeared to be located in the carboxy-terminal region of the MADS box proteins (Table S5 in Additional data file 1). This observation supports results from previous studies, where transcriptional activation capacity was often detected in the carboxy-terminal domain of plant MADS box proteins [10,21,29,30]. Subsequent analyses revealed that the identified motifs are underrepresented in the sequences of MADS box proteins that do not give auto-activation in yeast. Based on this, a decision tree model could be designed using those motifs that discriminate between auto-activating and non-auto-activating MADS box sequences, providing additional evidence for their role in transcriptional activation (Table S5 in Additional data file 1). As control, DILIMOT was used again to search for eventual overrepresented motifs in the set of MADS box proteins that do not give auto-activation in yeast. This search did not reveal any motif, consistent with their lack of transcriptional activation. When using the predicted auto-activation motifs to scan all proteins from the *Arabidopsis *genome, we found that these motifs are over two-fold overrepresented in transcription factors compared to all proteins, and that this overrepresentation is even higher (over four-fold) when analyzing proteins with at least two of the motifs present (Table S5 in Additional data file 1). This result provides additional validation for the putative role of the motifs in transcription activation. Note that one does not expect all transcription factors to be auto-activating, and, in addition, not all auto-activating transcription factors need to contain the same motifs.

**Figure 1:**
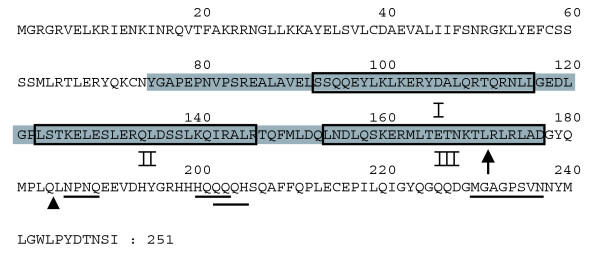
SEP3 protein sequence, domains and motifs. Predicted alpha helices are outlined and numbered (I-III) and the K-box (AA75-177, PFAM [84]) is shaded. Motifs predicted to be involved in transcriptional activation are underlined (NxNQ, HQxQ, QxQH, and MGxxxxxN). The arrow indicates the position at which SEP3ΔC1 stops (after amino acid 171) and the end of SEP3ΔC2 is indicated by an arrowhead (after amino acid 184).

**Figure 2:**
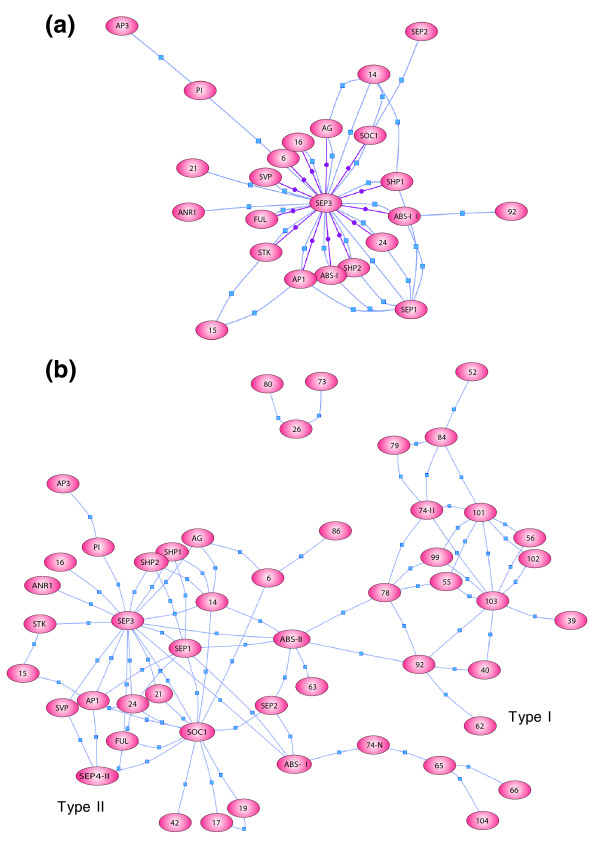
MADS box transcription factor interaction networks. **(a) **Visualization of a sub-network representing all SEP3 interactions and **(b) **the network representing all identified higher-order complexes. Proteins are indicated by ovals and interactions by lines. Purple lines indicate dimer formation and blue lines indicate ternary interactions. Ternary complexes are graphically represented in the network as a line between the protein that is expressed from the pAD-GAL4 vector and the protein expressed from the pARC352 vector (the dimer combination), and a line between the protein in the pARC352 vector and the pBD-GAL4 vector. Layout computed using the Pathway Studio 4.0 software (Ariadne Genomics, Inc., Rockville, MD, USA). Type I and type II MADS box protein sub-networks are indicated.

Figure 1 illustrates the putative transcriptional activation motifs in the SEP3 protein sequence. Previous studies have demonstrated that besides transcriptional activation capacity, ternary interaction determinants are also localized in the carboxy-terminal region of MADS box proteins [17], and, therefore, it was important to take this characteristic into account as well. Yang and Jack [31] performed an in-depth mapping of the domains involved in ternary complex formation between the B-function proteins and SEP3, and this study assigned an important role to the last predicted amphipathic alpha-helical structure at the border between the K-box and the carboxy-terminal region (Figure 1). Stimulated by these results, we used the web-based programs Paircoil [32] and Multicoil [33] to predict alpha-helical structures within the SEP3 protein. Based on these predictions and the identified putative activation domains, we designed two truncated SEP3 proteins lacking 80 and 67 amino acid residues at the carboxyl terminus, and named SEP3ΔC1 and SEP3ΔC2, respectively (Figure 1). The first truncated protein stops within the last predicted alpha helix, while the SEP3ΔC2 protein terminates directly after this predicted structural domain. Subsequently, the shortened proteins were fused to GAL4-BD and tested in yeast for auto-activation capacity, which appeared to be abolished in both cases. To investigate the ability of the two truncated SEP3 versions to form dimers and higher-order complexes, the previously identified heterodimer between AG and SEP3 [16] and the ternary complex between AG, STK and SEP3 [13] were tested in yeast. As expected, both SEP3ΔC protein versions were still able to dimerize with AG; however, only SEP3ΔC2 interacted with AG and STK in the yeast three-hybrid experiment, demonstrating once more the importance of the predicted alpha-helical structure at the end of the K-box for ternary protein interactions (helix III in Figure 1). Based on these observations, we reconstituted all known SEP3 dimers in yeast making use of the SEP3ΔC2 construct (Table S6 in Additional data file 1). This new collection of dimers was screened against all single MADS box proteins in a yeast three-hybrid assay, and reciprocally, the single SEP3ΔC2 protein fused to GAL4-BD was combined with the set of MADS domain dimers (Table S2 in Additional data file 1). This experiment yielded 59 additional higher-order complexes (Table S7 in Additional data file 1), including the known SEP3 ternary interactions (Table S1 in Additional data file 1). Figure 2a shows the sub-network representing all SEP3 interactions, whereas the overall network, including the complexes listed in Table S3 in Additional data file 1, is depicted in Figure 2b.

### SEP3 complex partners are co-expressed

A prerequisite for a biologically relevant protein-protein interaction *in planta *is coexistence of the proteins in the same cell and at the same moment during development. Therefore, the expression patterns of the genes encoding complex-forming MADS box proteins were compared using AtGenExpress data [34]. Note that a few MADS box genes are not presented on the ATH1 arrays used for AtGenExpress. For these particular MADS box genes, the AtTAX data were analyzed. This data set represent the results from whole genome tiling array hybridizations [35]. Unfortunately, no expression above background levels could be detected for most of the MADS box genes missing from the ATH1 arrays in the limited number of tissues tested on the tiling arrays. As a consequence, co-expression could not be confirmed for 16 out of the 106 identified complexes. Except for one complex, these are all complexes involving type I MADS box proteins, which are hardly studied. The co-expression analysis revealed that for almost 100% of the identified complexes containing type II MADS box proteins, the encoding genes have an overlap in expression in at least one tissue (Tables S3 and S7 in Additional data file 1). Remarkably, for type I proteins this was only 78%. This may reflect a real lack of co-expression, but, more likely, this is due to the low and very localized expression of a number of type I proteins [2,3,36-40], which makes the microarray data less reliable. For the few identified complexes consisting of combinations of type I and type II proteins, the expression patterns of the encoding genes appeared to overlap. The high percentage of co-expression (overall 95%) indicates that almost all identified complexes could potentially be formed *in planta*, although, for some of the genes, the expression levels were very low in the overlapping tissues. We also realize that these data are mRNA expression data and do not reflect protein levels; however, as far as is known, the spatial and temporal distribution of MADS domain proteins follows roughly the mRNA expression patterns [41,42]. Nevertheless, we can not exclude that non-cell autonomous action of MADS proteins plays a role and that some proteins are transported to adjacent cell layers and tissues. This has been shown, for instance, for the B-function MADS box proteins from *Antirrhinum *[43]. In Figure S1 in Additional data file 1 a comparison of expression patterns is presented for all gene combinations encoding putative ternary complex components for the complexes that contain the SEP3 protein.

### SEP3, AP3, and PI complex formation in living plant cells

**Figure 3:**
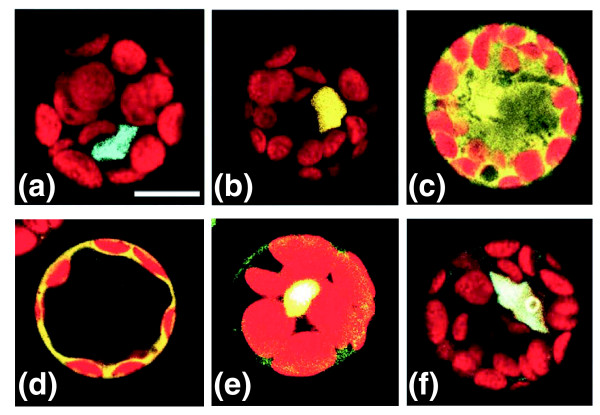
Localization of MADS box proteins in living cells. The MADS box proteins under study were fused to CFP or YFP and transiently expressed in *Arabidopsis *protoplasts. **(a) **PI-CFP; **(b) **SEP3-YFP; **(c) **AP3-YFP; **(d) **YFP-AP3; **(e) **YFP-AP3 + PI-CFP; **(f) **AP3-YFP + PI-CFP. Note that the proteins accumulate in a ring-like pattern at the position of the nucleolus. Scale bar = 10 μm.

To our surprise, a ternary complex was found in yeast between AP3, PI and SEP3, making use of full-length B-function proteins (Table S7 in Additional data file 1). Previous experiments revealed that the supposed heterodimer between AP3 and PI could not be detected in the yeast two-hybrid system when full-length proteins were used [16,44]. This strongly suggests that SEP3 can mediate the interaction between AP3 and PI in yeast. To investigate the behavior of these proteins in plant cells in more detail, we analyzed their interactions by fluorescence resonance energy transfer-fluorescence lifetime imaging microscopy (FRET-FLIM) in *Arabidopsis *leaf cells [23,45,46]. Initially, AP3, PI and SEP3 were carboxy-terminally labeled by enhanced cyan fluorescent protein (CFP) or enhanced yellow fluorescent protein (YFP) and transiently expressed in protoplasts, followed by confocal laser scanning microscopy for the analysis of their intracellular localization. Surprisingly, besides SEP3, PI was also nuclear localized, whereas the AP3 protein was found in both the nucleus and cytoplasm (Figure 3a-c). These localization results are not in agreement with previous intracellular localization data obtained for AP3 and PI in studies by McGonigle and colleagues [47], who observed that nuclear localization of the two B-function proteins occurs only when both proteins are simultaneously expressed. However, in their case, the GUS reporter was used and amino-terminally fused to the MADS box protein, followed by expression in onion epidermal cells, which might be the reason for the observed differences. It has been shown before that fusion of green fluorescent protein-like fluorophores to the amino terminus of MADS box proteins can influence their nuclear import [23,48]. To analyze whether there is a difference between amino- and carboxy-terminal labeling with respect to localization, AP3 and PI were also labeled with YFP at the amino terminus and transfected into protoplasts. In accordance with the results reported in the literature [47], most of the signal appeared to be localized in the cytoplasm in this case (Figure 3d); however, co-expression of the other B-function protein labeled at the carboxy-terminal results in a mainly nuclear localized signal for both proteins (Figure 3e) and the same result was obtained when both proteins were carboxy-terminally labeled (Figure 3f). Based on these observations, we decided to make use of carboxy-terminal fusions for all further experiments.

**Figure 4:**
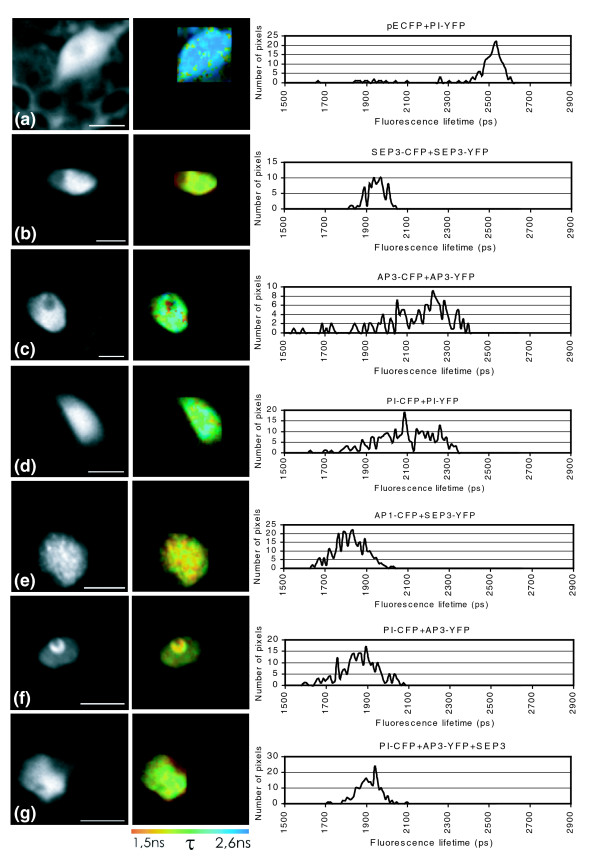
Analyses of MADS box protein interactions in protoplasts by FRET. *Arabidopsis *leaf protoplasts, co-expressing MADS box proteins fused to either CFP or YFP, were analyzed by FLIM, in order to detect FRET. One representative protoplast is shown for each analyzed combination. The left panels display the intensity channel, the middle panels show the fluorescence lifetime image of the same nucleus in a false color code, and the right panels depict histograms representing the distribution of fluorescence lifetime values over the nucleus. FLIM analysis on a protoplast transiently expressing **(a) **pECFP + PI-YFP (negative control); **(b) **SEP3-CFP + SEP3-YFP; **(c) **AP3-CFP + AP3-YFP; **(d) **PI-CFP + PI-YFP; **(e) **AP1-CFP + SEP3-YFP; **(f) **PI-CFP + AP3-YFP; **(g) **PI-CFP + AP3-YFP + SEP3. Scale bars = 10 μm.

FRET-FLIM was used to investigate the physical interaction of the labeled proteins in the leaf cells. The homodimer combinations 'SEP3-CFP + SEP3-YFP', 'PI-CFP + PI-YFP' and 'AP3-CFP + AP3-YFP' were analyzed first and 'PI-YFP + free CFP' was used as a negative control (Figure 4). Interestingly, a remarkable difference was detected among the proteins analyzed for homodimerization capacity. In the case of SEP3, a strong reduction of the fluorescence lifetime was observed over the entire nucleus, suggesting efficient homodimer formation (Figure 4b). In contrast, AP3 and PI showed only a strong reduction of fluorescence lifetime in particular sub-nuclear spots, which may represent more transient interactions (Figure 4c,d). Interaction in parts of the nucleus has been reported before for petunia MADS box proteins [23]. Currently, it is unclear whether these non-homogeneous interactions are biologically relevant; however, the ability of B-function proteins to homodimerize is supposed to be the ancestral status, which subsequently evolved into obligatory heterodimerization in the core eudicots [49]. In line with this, it could be that the homodimer interactions identified for the individual *Arabidopsis *B-function proteins by FRET-FLIM are remnants of their former ability to homodimerize, which has been almost lost during evolution. In a following experiment, we tested the supposed heterodimerization between the full-length PI and AP3 proteins in plant cells. Because no interaction was found between these two full-length proteins in yeast, the heterodimer between AP1 and SEP3 was added as a positive control [16]. As expected, the AP1-SEP3 combination showed a very strong reduction in fluorescence lifetime over the entire nucleus (Figure 4e). Interestingly, the combination AP3-PI also showed a strong FRET-FLIM signal demonstrated by a short fluorescence lifetime, suggesting that these proteins are able to form heterodimers in living plant cells (Figure 4f). Remarkably, this combination always resulted in a strong accumulation of fluorescent signal in a ring-like pattern at the position of the nucleolus (Figures 3f and 4f), a phenomenon that was never observed for any other combination of MADS box proteins tested.

Subsequently, the effect of SEP3 on the AP3-PI heterodimer was analyzed by FRET-FLIM to gain insight into higher-order complex formation. For this purpose the occurrence of FRET was measured between PI-CFP and AP3-YFP in the presence of a non-labeled SEP3 protein. The addition of SEP3 appeared to have a strong effect on the localization of the PI and AP3 proteins: instead of localization at the nucleolus (Figure 4f), the AP3 and PI protein interaction appeared to be more equally distributed over the nucleus in the presence of SEP3 (Figure 4g). Furthermore, a short fluorescence lifetime could be observed over the entire nucleus, although the drop in fluorescence lifetime was less strong than in the absence of SEP3 (Figure 4f). An explanation for this could be that SEP3, which is supposed to bind to the carboxy-terminal regions of AP3 and PI, interferes with the optimal positioning of CFP and YFP for a high FRET efficiency.

## Discussion

### Plant MADS domain protein higher-order complex formation

MADS box transcription factors play essential roles during the plant lifecycle and can be characterized as the architects of plant development. Their specific functioning is mainly determined by direct physical protein-DNA and protein-protein interactions (reviewed in [45,50]). Besides the formation of dimers, the well studied type II floral organ identity MADS box proteins [51] are supposed to form multimeric protein complexes consisting of three to four different MADS box proteins (for example, [8,17,21]). Remarkably, the majority of higher-order complexes known to date contains at least one protein belonging to the 'E-function' class, which is represented by the SEP proteins in *Arabidopsis *[7]. It was unknown whether assembly into these large complexes is a common molecular mechanism that mediates plant MADS box transcription factor functioning, or whether this is only characteristic for the 'ABC-function' proteins and, in particular, for 'E-function' proteins. Therefore, we performed a large-scale yeast three-hybrid analysis for members of the *Arabidopsis *MADS box transcription factor family. Although this study was not comprehensive due to technical limitations of the screen, many novel complexes could be identified for both type I and type II MADS box transcription factors. In the initial screen with the full-length proteins, more complexes were identified that exclusively consist of type II proteins (25) than complexes with only type I proteins (15), while the *Arabidopsis *genome encodes more proteins belonging to the latter class. Whether this difference in the capacity to assemble into multimeric complexes between these two groups is due to differences in protein structure and reflects their biological functions needs more thorough investigations by alternative methods. The fact that type I proteins lack a K-box, which has been shown to be an important mediator for dimerization and higher-order complex formation [31,44], could explain the observed differences. Nevertheless, coiled-coil structures have been predicted within the carboxy-terminal region of type I proteins [2] and these structural motifs are well-known molecular recognition structures [52] that potentially can be involved in type I complex formation.

In the previous two-hybrid screen from De Folter and colleagues [16], interactions between type I and type II MADS box proteins were observed, although rare. In the current three-hybrid screen also only a few complexes (7) were found that contain both type I and type II proteins, though the genes encoding these interacting proteins are co-expressed (Table S3 in Additional data file 1). The presence of these interactions suggests that they arose before the duplication that gave rise to the two lineages, which happened before the divergence of plants and animals [51]. Alternatively but less likely, these hybrid interactions were acquired after the birth of the type I and II MADS box lineages. Interestingly, the interaction networks of the type I and type II proteins are clearly separated (Figure 2b), which may reflect the different functions these proteins play in plants. Most type II proteins are involved in identity specification and phase changes, while recent studies on type I genes [2,3,36-40] support the notion that they play an important role in gametophyte and embryo development. The inter-lineage interactions between the type I and II sub-networks may link the different roles these MADS box proteins play. In this respect it is interesting to notice that five out of seven 'type I-type II' interactions contain either the type II proteins ARABIDOPSIS BSISTER (ABS) or AG; both proteins are important for gametophyte and seed development in *Arabidopsis *[20,27,53]. The *ABS *gene encodes two proteins, ABS-I and ABS-II, which are derived through alternative splicing [20]. The yeast three-hybrid experiments revealed that both proteins multimerize with type I proteins, but with a difference in specificity. Besides these differences, novel and distinctive interactions with type II proteins were also found for the two ABS proteins, which had not been identified in previous studies [20,27]. These differences in interaction specificity probably explain the observation that only the long splice form (ABS-I) can complement the endothelium defects in the *abs *mutant [20]. In contrast to ABS-II, the ABS-I protein is able to form a ternary complex with AGAMOUS-LIKE16 (AGL16)-SEP3, PI-SEP3, AGL74N-SEP2 and SEP1-SEP2. Except for 'AGL74N-SEP2-ABS-I', co-expression of the genes encoding these interacting proteins in carpels and young pistils containing seeds has been detected [34]. Unfortunately, detailed information about expression in the ovule and function of these ABS-I specific interaction partners is missing, leaving the question of whether one of these novel complexes is responsible for the functional discrepancy between ABS-I and ABS-II unanswered.

### Expression of the genes encoding complex members

In general, co-expression of genes encoding interaction partners may give clues about a common function for the proteins involved. For example, members of the MIKC* sub-clade (also known as Mδ [2]) are specifically expressed during pollen formation and the encoded proteins form higher-order complexes with other members of this sub-clade, suggesting that they play an important role during pollen development [54]. However, a lack of a large expression overlap *in planta *does not necessarily mean that we are dealing with a false positive protein interaction. Note that, for example, the AG-SEP3 dimer interacts with a set of ternary interacting factors that overlap in expression pattern with the dimerization partners in distinct tissues, or during particular stages of development only (Tables S3 and S7 and Figure S1 in Additional data file 1). Complexes were also identified for proteins that show no obvious overlap in their corresponding mRNA expression patterns, as, for example, complexes consisting of the floral activators AGL24 [55], SUPRESSOR OF OVEREXPRESSION OF CONSTANS1 (SOC1) [56], and the AGL17 or AGL19 proteins, which are both encoded by genes preferentially expressed in roots [57,58]. However, recent functional analyses of AGL17 [59] and AGL19 [58] revealed that these proteins are also inducers of flowering and share this function with their putative complex partners. Besides the expression in roots, both *AGL17 *and *AGL19 *show low expression in above-ground vegetative parts [58,59], which probably results in sufficient molecules for complex formation and subsequent activation of flowering in the shoot apical meristem. Furthermore, it is known that the expression levels of *AGL24 *[60], *SOC1 *[61], and *AGL17 *[59] are coordinately up-regulated by CONSTANS (CO) and, hence, that these MADS box genes act downstream of this protein in the photoperiodic flowering pathway. Based on all these findings, we hypothesize that the specific higher-order complex formation between these MADS box proteins is an important mechanism for the functioning of these proteins in the regulation of flowering time. Notably, similar kinds of complexes have been found for a couple of other related and preferentially root-expressed MADS box proteins (AGL14, AGL21 and AGL42) [57,62,63], whose functions are unknown. From the genes encoding these proteins, *AGL42 *is strongly up-regulated upon a switch from short day to long day conditions, as is the case for *SOC1 *and *AGL24 *[64]. Based on the common complex formation partners identified in this study, we may speculate that the AGL42 protein also plays a role in floral induction.

### The importance of SEP proteins for multimerization

SEP proteins seem to be important mediators of higher-order complex formation and, therefore, we have focused on the capacity of the SEP3 protein to form multimeric complexes. In the dedicated yeast three-hybrid screen with the carboxy-terminally truncated SEP3 protein, known SEP3 ternary complexes were confirmed, showing that the conditions of our yeast three-hybrid assay permit the detection of these ternary interactions. To our surprise, the screen with the truncated SEP3 protein more than doubled the total number of identified ternary MADS box protein complexes. Despite the fact that the number of ternary interactions found in this study resembles most likely only a small proportion of the potential higher-order complexes present in *Arabidopsis*, this result reveals an important role for SEP3 in MADS box protein complex formation. Therefore, the SEP3 protein can be regarded as a 'glue' that mediates the assembly of MADS box transcription factor complexes and is functional as a hub in the MADS box transcription factor interaction network. We may hypothesize that the other SEP proteins have a similar specificity for higher-order complex formation, knowing that there is functional redundancy within this clade of MADS box proteins [7,11]. In line with this idea, the comprehensive yeast two-hybrid screening performed by us showed similar binary interactions for SEP1 and SEP3 [16]. However, SEP2 and SEP4-I/II seem to have a number of different dimerization partners in yeast; also in the yeast three-hybrid screen presented in this report, specific complexes were identified for SEP2 and SEP4-II that could not be found for SEP3. Together, this suggests that the functional redundancy present in the *Arabidopsis *SEP clade is not complete and, hence, that some of the SEP proteins have gained or maintained specific interactions and functions that are not shared by the other members of the family. A similar comprehensive approach as followed in this study for SEP3, consisting of mapping the auto-activation domain and performing the three-hybrid screen with mutated or truncated clones, would be needed for each individual SEP protein to elucidate their specific ternary complex formation capacities. Regardless of the outcome of such an experiment, however, it is clear from the genetic studies that besides small differences, there is overlap between the functions of the SEP proteins in the inner three whorls of the flower, which means that the different SEP proteins should have the capacity to form complexes with at least some common MADS box partners. Assuming that SEP3 is the 'glue' for higher-order complex formation in the inner three floral whorls, the question arises as to which SEP protein functions as 'glue' during the vegetative stage of development. *SEP4 *is expressed early during development in the green parts of the plant, in contrast to *SEP3 *[34], though at relatively low levels. Because of this, it may also be possible that another type II MADS box protein is functional as a 'glue' protein during the vegetative stage. In this respect, SOC1 is a good candidate, because it has the right spatial expression pattern and a large number of two-hybrid interaction partners like the SEP proteins. It functions as a hub in the two-hybrid network [16] and, more importantly, this protein is incorporated in ternary complexes almost as frequently as SEP3 (Tables S3 and S7 in Additional data file 1).

### Biological functions of ternary SEP3 MADS box protein complexes

**Figure 5:**
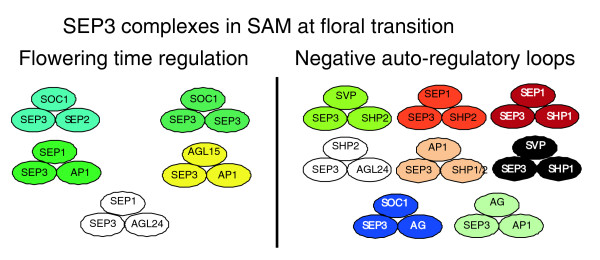
SEP3 ternary complexes that, based on expression patterns of the genes encoding the involved proteins, might be formed in the shoot apical meristem (SAM) at the moment of the phase switch between vegetative and generative development. Taking into account known functions for some of these proteins, the complexes have been categorized in two classes; one for complexes supposed to be involved in regulating the timing of flowering, and one for complexes that might function in negative auto-regulatory loops. Our hypothesis is that complexes from this latter group are essential for the repression of the genes involved in timing of flowering in the floral organs.

Studies performed previously revealed the importance of SEP proteins present in ternary and quaternary floral organ identity complexes [8,9] and recent *in planta *protein localization studies showed co-localization of the 'ABC' proteins in accordance with the 'ABC model' [42]. Besides these interactions with other ABC-function MADS box proteins, our results have shown that the SEP3 protein is potentially incorporated in complexes with MADS box proteins involved in the regulation of flowering time, such as SOC1 [56], AGL24 [55], SHORT VEGETATIVE PHASE (SVP) [65], and AGL15 [66] (Figure 5). These interactions suggest that the SEP3 protein also functions in the transition to flowering, which is in line with observations in a study by Pelaz and colleagues [67], who obtained an enhanced early flowering phenotype for *Arabidopsis *plants ectopically expressing both *AP1 *and *SEP3 *when compared to plants over-expressing *AP1 *alone. Expression of the SEP3 protein could not be detected in vegetative tissues; however, the protein is present at low levels in the inflorescence meristem [42]. SEP3 probably performs this early function redundantly with SEP4, which, in contrast to SEP3, is expressed during the vegetative stage of development and is also able to form a couple of ternary complexes with the flowering time MADS box proteins. In addition or alternatively, ternary complexes consisting of MADS box proteins involved in regulation of flowering time and floral organ identity proteins (for example, SEP3, AG, AP1) could function in negative auto-regulatory feed-back mechanisms (Figure 5). De Folter and colleagues [16] hypothesized that the expression of genes encoding floral inducing MADS box proteins is down-regulated in the floral organ primordia by a negative auto-regulatory loop involving dimerization of the encoded proteins with the MADS box proteins functioning in floral organ development [16]. Recently, the research group of Yu showed that the floral meristem identity protein AP1 is involved in the down-regulation of the flowering time genes *SOC1*, *SVP *and *AGL24 *[68]. Based on our results, it is tempting to speculate that down-regulation of these flowering time genes is mediated by a negative feed-back loop, in which both the flowering proteins and SEP3 are involved (Figure 5). In line with this, *in situ *hybridization analyses for flowering time genes in wild-type plants show hardly any signal during later stages of flower development [68,69]. However, in mutant backgrounds of floral organ identity MADS box genes, such as *ap1*, *ag*, and *sep1*/*sep2*/*sep3*, ectopic expression of these flowering time genes is obtained in floral tissues [68,69]. Although this gives strong evidence for the supposed negative auto-regulatory loops, further studies are required to support the hypothesis that higher-order complexes are essential for this function.

**Figure 6:**
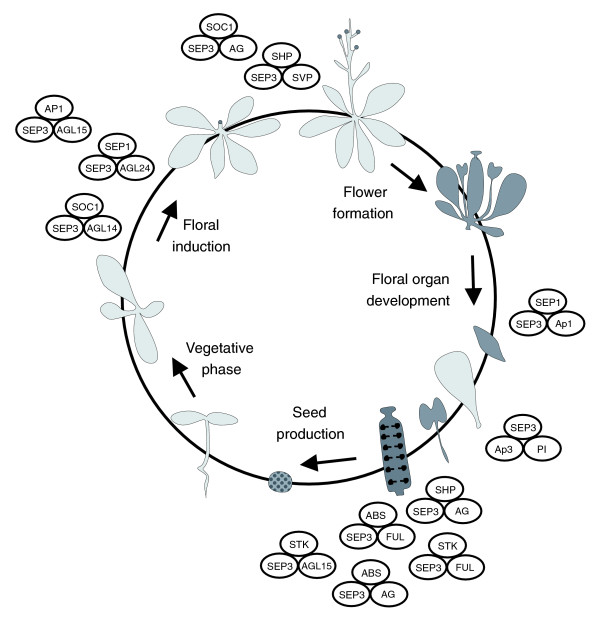
Putative function of SEP3 complexes during plant development. Some of the identified higher-order SEP3 complexes have been placed in the *Arabidopsis *life cycle at the stage in which they are supposed to be functional. For all the indicated complexes the genes encoding the proteins are co-expressed in a particular tissue or developmental stage (Tables S3 and S7 and Figure S1 in Additional data file 1). Note, that the graphical representation probably does not reflect the real stoichiometry of the complexes. It is possible, for example, that proteins are present as homodimers in the complexes.

It is difficult to assign a biological role for some of the other ternary SEP3 complexes identified in our study because no information is available about the functions of the individual proteins. Furthermore, many proteins may have multiple functions throughout the life cycle of a plant and, therefore, late functions can be masked by early functions in genetic studies. The expression of MADS box genes late during development of the floral organs [42] and the late functions identified for, for example, B-function MADS box proteins [70,71] and AG [72] demonstrate that these transcription factors are multi-tasking and play a role during further differentiation of the floral organs. These various functions are reflected in the different complexes formed by such a MADS box protein, each supposed to regulate a specific set of target genes. SEP3 is part of many complexes and, therefore, may bind to different target genes controlling distinct developmental pathways. Because the *sep1 sep2 sep3 *triple mutant produces only sepals in the flower [7], it is difficult to predict *SEP3 *functions at later flowering stages, but, based on its expression pattern and our interaction data, we could assign a role for this protein as a key regulator in many developmental processes (Figure 6). For instance, the protein complex consisting of SEP3, STK and AG is involved in ovule identity specification [13], while a combination with the integument and seed coat-specific protein ABS may be required for the subsequent steps in seed development [20].

### Molecular function of SEP3 in ternary MADS box protein complexes

As shown by yeast-based and FRET-FLIM studies, the ternary factor SEP3 is able to stabilize dimeric interactions and to affect the subcellular localization of its interaction partners. Stabilization of a MADS box transcription factor dimer by a ternary factor has been shown in petunia before [23] and may be a general function for ternary MADS box factors. The effect of SEP3 on AP3-PI localization could play an important role in the temporal storage or, alternatively, in the activation of this specific MADS box protein dimer. Recently, it has been shown that the mammalian basic helix-loop-helix transcription factor Hand1 is sequestered in the nucleolus due to interaction with a co-factor, and that the release of this protein from the nucleolus is essential for its activation [73]. Similar mechanisms may play a role in activating particular plant MADS box proteins, such as AP3 and PI. The question remains if this is the only function for ternary factors, like SEP3, in higher-order complexes. SEP3 appears to contain a strong transcription activation domain and, based on this, it has been hypothesized that an important function of this ternary factor is to add transcriptional activity to multimeric transcription factor complexes [10]. This might be true, but, at least in the case of the complex AP3-PI-SEP3, the SEP3 protein is doing more, because plants with constitutive overexpression of AP3 and PI fused to the VP16 trans-activation domain do not show homeotic changes of cauline leaves into floral organs (K Goto, personal communication). In contrast, the constitutive expression of AP3 and PI in combination with SEP3 gives conversions of cauline leaves into petals [8,9]. Although the combination of constitutive *AP3 *and *PI-VP16 *expression is sufficient to activate the positive auto-regulatory loop for the B-function MADS box genes - that is, it activates the *AP3 *promoter [10] - it is not sufficient for the regulation of all AP3-PI target genes that are essential for petal development. In conclusion, SEP3 can change the sub-nuclear localization of the AP3-PI heterodimer and probably this is crucial for petal development. Furthermore, Egea-Cortines and colleagues [17] have shown that ternary complexes bind more strongly to the consensus CArG-box in DNA sequences than MADS box protein dimers. SEP3 in a multimeric complex may facilitate the protein-DNA interaction, either by stabilizing the dimer or by direct binding to the DNA and providing specificity. In the latter case, the DNA will bend and the transcription complex will bind to two binding sites at a short distance from each other. In addition, ternary complex formation may play a role in the recruitment of co-factors. Recent studies have shown that the MADS box proteins AGL24 and SVP are not able to interact with the LEUNIG-SEUSS co-repressor complex, although interaction between these proteins could be mediated by the AP1 protein [74]. In a similar way, SEP3 enables the interaction between MADS box proteins involved in ovule development (for example, STK) and the BELL1 homeodomain factor [75].

## Conclusions

This study yielded a collection of potential multimeric MADS domain protein complexes in which SEP3, the 'glue protein', plays a central role. Besides the initial steps of floral organ formation, this protein seems to function in various other plant developmental processes via multimerization (Figures 5 and 6). Higher-order complex formation of MADS domain proteins appears to be a common process and provides these transcription factors with unique attributes to function in a specific manner, such as the possibility to change interaction stability, localization of the proteins, and their DNA binding specificity. Combining protein interaction analyses as performed in this study and co-expression analyses provides complementary functional information about MADS transcription factors, in particular when mutant phenotypes are missing due to redundancy or when the proteins are involved in multiple developmental processes, as is the case for SEP3.

## Materials and methods

### Plant material

Protoplasts were obtained from *Arabidopsis thaliana *Col-0 leaves, which were grown under normal greenhouse conditions (16/8 h light/dark, 22°C), according to Aker and colleagues [76].

### Plasmid constructions

For the yeast three-hybrid experiments two new *SEP3 *Gateway entry clones were generated, encoding the carboxy-terminally truncated versions of this protein. The first clone, designated *SEP3*ΔC1, encodes SEP3 lacking the last 80 amino acids of the carboxyl terminus and the second clone, *SEP3*ΔC2, encodes the SEP3 protein lacking 67 amino acids at its carboxyl end. The truncated coding regions were obtained by PCR and a new stop codon was included in the reverse primer. Subsequently, the PCR fragments were cloned into pCR8/GW/TOPO (Invitrogen, Carlsbad, CA, USA), followed by sub-cloning via a Gateway LR reaction into pBDGAL4 (pDEST32; Invitrogen) and the Gateway compatible pTFT1 yeast expression vector (pARC352) [28]. For the *in vivo *localization and interaction studies, the coding region of the MADS box genes *APETALA1 *(*AP1*), *APETALA*3 (*AP3*), *PISTILLATA *(*PI*), *AGAMOUS *(*AG*), *SEP3 *and *SEP*3ΔC1, were cloned as Gateway entry clones without stop codons (pCR8/GW/TOPO; Invitrogen), in order to allow carboxy-terminal fusions. The obtained entry clones were recombined into the Gateway compatible destination vectors pARC971 and pARC428 from which expression is driven by the constitutive CaMV35S promoter and that contain the coding regions of the fluorophores CFP and YFP, respectively [23]. Furthermore, amino-terminal fusions were made for AP3 and PI. In this case, the destination vector was pK7WGY2,0 from the VIB collection [77], containing the coding region of the YFP molecule. AP3 and PI entry clones including stop codons were taken from the REGIA collection [2,16]. All plasmids were controlled by sequence analyses (DETT sequence kit; Amersham, Sunnyvale, CA, USA).

### Yeast three-hybrid screen

Transformations of yeast strain PJ69-4, mating type A and α [78], were done as described by the laboratory of Gietz [79]. Triple combinations of MADS box proteins in yeast were obtained by robotized mating between individual matα yeast cultures containing 'pBD-GAL4-MADS' vectors [16] and matA yeast cultures containing the MADS dimers (Tables S2 and S6 in Additional data file 1), following the protocol described before [16]. The mated yeast was grown for 2 days at 30°C on plates with synthetic dropout medium without leucine (L), tryptophan (W) and adenine (A), to select for the presence of all three plasmids. Subsequently, some yeast material was resuspended in 50 μl sterile water, in a 96-well micro-titer plate. Aliquots (5 μl) of these suspensions were spotted onto synthetic dropout medium plates lacking the amino acids L, W, A and histidine (H) and supplemented with 1 mM 3-amino-1,2,4-triazole (3-AT) in a grid of 96 spots, to select for protein interactions. These plates were incubated at 20°C for 7 days before scoring of yeast growth. All positives due to dimerization between two of the three proteins, and/or auto-activation by the MADS box protein expressed from the pBD-GAL4 vector, or its dimerization partner in the pARC352 vector, were discarded based on our knowledge from the large-scale yeast two-hybrid experiment [16]. For all remaining positive combinations the mating was repeated and the interaction confirmed by spotting onto plates with synthetic dropout medium lacking amino acids L, W, A, H and supplemented with 1, 5, or 10 mM 3-AT. In parallel, a LacZ assay [80] was performed to test for the activation of this second reporter gene. All combinations that were scored at least two times positive and for both the HIS and LacZ selection markers, were selected as true positives.

### Fluorescence microscopy in living cells

*Arabidopsis *leaf protoplasts were transfected as described by Aker and colleagues [76]. Plasmid DNA (15-30 μg) was used and the protoplasts were incubated overnight at 25°C before imaging. Images were made using a confocal laser microscope 510 (Carl Zeiss, Jena, Germany). The Argon laser was used to excite at 458 and 514 nm for CFP and YFP, respectively. Fluorescence was detected through a band pass filter of 470-500 nm for CFP and 535-590 nm for YFP [76].

### FRET-FLIM measurements in living cells

FRET-FLIM analyses were done in *Arabidopsis *protoplasts as described before [23,81]. The donor fluorescence lifetime was measured on the central part of the nucleus of each single cell, pixel by pixel, and at least ten cells were analyzed per combination in three independent experiments. The donor lifetime of CFP was fixed at 2.6 ns for all further analyses. Images were acquired by using the Becker and Hickl 1 SPC 830 module, and SPC image 2.8 software was used for the data analyses (Becker and Hickl, Berlin, Germany).

### Prediction of transcriptional activation

DILIMOT [82] was applied using default parameters (maximum motif length 8, number of fixed positions 3, minimal number of motifs in dataset 3) on all 19 sequences of MADS box proteins showing auto-activation in yeast [16]. Subsequently, using ps_scan [82], it was confirmed that these motifs occur much less often in other MADS box protein sequences. To obtain further insight into the role of these motifs, a decision tree model was built (using the function 'tree' in the software package R) with class indicator 'auto-activation' or 'no auto-activation' and variables describing occurrence of each motif in the sequences. This analysis selected five motifs out of the ten motifs returned by DILIMOT (Table S5 in Additional data file 1) and resulted in a model with over 80% accuracy, 80% specificity and 50% coverage. The accuracy is the overall percentage of correct predictions and the specificity indicates the percentage of predicted auto-activating proteins for which auto-activation was identified in yeast. The coverage gives the percentage of experimentally detected auto-activating proteins that were also predicted to give auto-activation.

### Co-expression analysis

The developmental set of the AtGenExpress expression atlas [34] was analyzed for expression of MADS box genes, as previously described [16]. A threshold of log_2 _≥ 4 was applied to identify overlap in tissues with expression of genes. For genes not expressed in the AtGenExpress expression atlas (*AGL13*, *AGL61*, *AGL92*, *AGL96*, and *AGL103*) other publicly available expression data were used [2,35,83].

## Abbreviations

3-AT: 3-amino-1,2,4-triazole; ABS: ARABIDOPSIS BSISTER; AD: activation domain; AG: AGAMOUS; AGL: AGAMOUS-LIKE; AP: APETALA; BD: binding domain; CFP: enhanced cyan fluorescent protein; FLIM: fluorescence lifetime imaging; FRET: fluorescence resonance energy transfer; PI: PISTILLATA; SEP: SEPALLATA; SOC: SUPRESSOR OF OVEREXPRESSION OF CONSTANS; STK: SEEDSTICK; SVP: SHORT VEGETATIVE PHASE; YFP: enhanced yellow fluorescent protein.

## Authors' contributions

RGHI supervised the projects in which the work was carried out, performed part of the cloning, set-up the yeast three-hybrid screening method, and wrote the manuscript together with INT. In addition, INT performed the localization and FRET-FLIM experiments. SdF performed the network and co-expression analyses and was involved in scientific discussions. All yeast vectors and the yeast collections and glycerol stocks were prepared by AS. ADJvD performed the bioinformatics predictions for transcription activation domains and JBL performed the yeast three-hybrid screenings. The FRET-FLIM experiments were supervised by JWB and he performed a few experiments of this type. GCA contributed to all scientific discussions and critically revised the manuscript.

## Additional data files

The following additional data are available with the online version of this paper. Additional data file 1 contains Tables S1-S7 and Figure S1. Table S1 lists all higher-order complexes for *Arabidopsis *MADS domain proteins reported in the literature. Table S2 provides an overview of all MADS domain protein dimers that have been generated in yeast. Table S3 presents the higher-order complexes that were identified in the initial yeast three-hybrid screening. Table S4 lists the few dimers that could be extracted from the dataset in Additional file 3 and that were not identified before in the comprehensive two-hybrid screening [16]. Table S5 gives information about the prediction of transcription activation domains in the MADS protein sequences. Table S6 lists the yeast collection containing all SEP3ΔC2 dimers. Table S7 lists all ternary complexes that were identified using this SEP3ΔC2 collection. Figure S1 shows the data of the co-expression analysis for genes encoding interacting MADS domain proteins.

## Supplementary Material

Additional data file 1 Table S1 lists all higher-order complexes for *Arabidopsis *MADS domain proteins reported in the literature. Table S2 provides an overview of all MADS domain protein dimers that have been generated in yeast. Table S3 presents the higher-order complexes that were identified in the initial yeast three-hybrid screening. Table S4 lists the few dimers that could be extracted from the dataset in Additional file 3 and that were not identified before in the comprehensive two-hybrid screening [16]. Table S5 gives information about the prediction of transcription activation domains in the MADS protein sequences. Table S6 lists the yeast collection containing all SEP3ΔC2 dimers. Table S7 lists all ternary complexes that were identified using this SEP3ΔC2 collection. Figure S1 shows the data of the co-expression analysis for genes encoding interacting MADS domain proteins. Click here for file
