# Supplementary material for: SEPALLATA3: the 'glue' for MADS box transcription factor complex formation
Source: Genome Biol. 2009 Feb 25;10(2):R24. doi: 10.1186/gb-2009-10-2-r24 (PMC2688274; doi:10.1186/gb-2009-10-2-r24)
Supplement: Additional data file 1 — Table S1 lists all higher-order complexes for Arabidopsis MADS domain proteins reported in the literature. Table S2 provides an overview of all MADS domain protein dimers that have been generated in yeast. Table S3 presents the higher-order complexes that were identified in the initial yeast three-hybrid screening. Table S4 lists the few dimers that could be extracted from the dataset in Additional file 3 and that were not identified before in the comprehensive two-hybrid screening [16]. Table S5 gives information about the prediction of transcription activation domains in the MADS protein sequences. Table S6 lists the yeast collection containing all SEP3ΔC2 dimers. Table S7 lists all ternary complexes that were identified using this SEP3ΔC2 collection. Figure S1 shows the data of the co-expression analysis for genes encoding interacting MADS domain proteins. [file gb-2009-10-2-r24-S1.doc]

# Additional data file 1

**Table S1:Ternary and quaternary complexes of Arabidopsis MADS box proteins known from literature.**

PISTILLATA (PI), APETALA3 (AP3), SEPALLATA3 (SEP3), APETALA1 (AP1), AGAMOUS (AG), SEEDSTICK (STK), SHATTERPROOF1 (SHP1), SHATTERPROOF2 (SHP2), ARABIDOPSIS B_sister_ splice form I (ABS-I), ARABIDOPSIS B_sister_ splice form II (ABS-II). ND = not determined.

| **Complex forming proteins** | **Confirmed in this study (Y/N)** | **Reference** |
| --- | --- | --- |
| PI + AP3 + SEP3 | Y | [1] |
| PI + AP3 + AP1 | N | [1] |
| PI + AP3 + SEP3 + AG | ND | [1] |
| AG + SEP3 + AG | Y | [2] |
| AG + SEP3 + STK | Y | [2] |
| AG + SEP3 + SHP1 | Y | [2] |
| AG + SEP3 + SHP2 | Y | [2] |
| STK + SEP3 + STK | Y | [2] |
| STK + SEP3 + SHP1 | Y | [2] |
| STK + SEP3 + SHP2 | Y | [2] |
| SHP1 + SEP3 + SHP1 | Y | [2] |
| SHP1 + SEP3 + SHP2 | Y | [2] |
| STK + SEP3 + ABS-I | Y | [3, 4] |
| SHP1 + SEP3 + ABS-I | Y | [4] |
| SHP2 + SEP3 + ABS-I | Y | [4] |
| AG + SEP3 + ABS-I | Y | [3] |
| AG + SEP3 + ABS-II | Y | [3] |
| STK + SEP3 + ABS-II | Y | [3] |
| SHP1 + SEP3 + ABS-II | Y | [3] |
| SHP2 + SEP3 + ABS-II | Y | [3] |

**Table S2: All Arabidopsis MADS box transcription factor dimers that have been generated in yeast strain PJ69-4A (mating type A).**

| ***Dimer number*** | ***pADGAL4 insert*** | ***pTFT1 insert*** | ***Dimer number*** | ***pADGAL4 insert*** | ***pTFT1 insert*** |
| --- | --- | --- | --- | --- | --- |
| **1** | AGL103 | AGL102 | **139** | AGL73 | AGL101 |
| **2** | AGL103 | AGL55 | **140** | AGL26 | AGL73 |
| **3** | AGL103 | AGL56 | **141** | AGL73 | AGL43 |
| **4** | AGL104 | AGL65 | **142** | AGL52 | AGL73 |
| **5** | AGL14 | AGL16 | **143** | AGL73 | AGL53 |
| **6** | AGL15 | SHP1 | **144** | AGL77 | AGL73 |
| **7** | AGL15 | STK | **145** | AGL73 | AGL78 |
| **8** | AGL15 | AGL15 | **146** | AGL73 | AGL89 |
| **9** | AGL15 | AGL16 | **147** | AGL103 | AGL74-II |
| **10** | AGL15 | SOC1 | **148** | AGL74-II | AGL78 |
| **11** | AGL15 | SVP | **149** | AGL74N | SHP1 |
| **12** | AGL15 | AGL24 | **150** | AGL74N | CAL |
| **13** | AGL15 | SHP2 | **151** | AGL74N | AGL101 |
| **14** | AGL15 | AGL6 | **152** | AGL103 | AGL74N |
| **15** | AGL15 | AP1 | **153** | AGL74N | STK |
| **16** | AGL15 | AG | **154** | AGL74N | AGL12 |
| **17** | AGL16 | SHP1 | **155** | AGL74N | AGL13 |
| **18** | AGL16 | STK | **156** | AGL74N | AGL17 |
| **19** | AGL16 | AGL12 | **157** | AGL26 | AGL74N |
| **20** | AGL16 | AGL16 | **158** | AGL74N | FLM |
| **21** | AGL16 | AGL17 | **159** | AGL74N | ABS-I |
| **22** | AGL16 | AGL21 | **160** | ABS-II | AGL74N |
| **23** | AGL16 | ANR1 | **161** | AGL74N | SEP4-I |
| **24** | AGL16 | SHP2 | **162** | AGL74N | SEP4-II |
| **25** | AGL16 | AGL6 | **163** | AGL74N | AGL42 |
| **26** | AGL16 | AGL63 | **164** | AGL74N | AGL43 |
| **27** | AGL16 | AP1 | **165** | AGL74N | AGL49 |
| **28** | SHP1 | AGL13 | **166** | AGL74N | SHP2 |
| **29** | SOC1 | SHP1 | **167** | AGL52 | AGL74N |
| **30** | SOC1 | CAL | **168** | AGL74N | AGL55 |
| **31** | SOC1 | AGL12 | **169** | AGL74N | AGL56 |
| **32** | SOC1 | AGL13 | **170** | AGL74N | AGL6 |
| **33** | SOC1 | AGL14 | **171** | AGL74N | AGL65 |
| **34** | SOC1 | AGL16 | **172** | AGL74N | AP1 |
| **35** | SOC1 | AGL17 | **173** | AGL74N | AGL71 |
| **36** | SOC1 | AGL19 | **174** | AGL74N | AGL72 |
| **37** | SOC1 | SOC1 | **175** | AGL77 | AGL74N |
| **38** | SOC1 | SVP | **176** | AGL74N | AGL78 |
| **39** | SOC1 | SEP4-I | **177** | AGL74N | AGL82 |
| **40** | SOC1 | SEP4-II | **178** | AGL74N | AGL87 |
| **41** | SOC1 | SEP2 | **179** | AGL74N | AGL90 |
| **42** | SOC1 | AGL42 | **180** | AGL74N | AGL96 |
| **43** | SOC1 | ANR1 | **181** | AGL74N | AG |
| **44** | SOC1 | SHP2 | **182** | AGL78 | AGL102 |
| **45** | SOC1 | AGL6 | **183** | AGL78 | AGL55 |
| **46** | SOC1 | AP1 | **184** | AGL78 | AGL56 |
| **47** | SOC1 | AGL71 | **185** | AGL78 | AGL6 |
| **48** | SOC1 | FUL | **186** | AGL78 | AGL74-II |
| **49** | AGL21 | SHP1 | **187** | AGL77 | AGL78 |
| **50** | AGL21 | STK | **188** | AP1 | AGL6 |
| **51** | AGL21 | AGL12 | **189** | AGL62 | AGL80 |
| **52** | AGL21 | AGL13 | **190** | AGL82 | AGL102 |
| **53** | AGL21 | AGL15 | **191** | AGL83 | AGL53 |
| **54** | AGL21 | AGL16 | **192** | AGL101 | AGL84 |
| **55** | AGL21 | AGL17 | **193** | AGL26 | AGL84 |
| **56** | AGL21 | AGL19 | **194** | AGL43 | AGL84 |
| **57** | AGL21 | SOC1 | **195** | AGL52 | AGL84 |
| **58** | AGL21 | AGL21 | **196** | AGL53 | AGL84 |
| **59** | AGL21 | SVP | **197** | AGL76 | AGL84 |
| **60** | AGL21 | AGL24 | **198** | AGL77 | AGL84 |
| **61** | AGL21 | SEP2 | **199** | AGL78 | AGL84 |
| **62** | AGL21 | AGL42 | **200** | AGL89 | AGL84 |
| **63** | AGL21 | ANR1 | **201** | AGL86 | AGL23 |
| **64** | AGL21 | SHP2 | **202** | AGL86 | AGL28 |
| **65** | AGL21 | AGL6 | **203** | AGL86 | SEP4-II |
| **66** | AGL21 | AP1 | **204** | AGL86 | AGL40 |
| **67** | AGL21 | FUL | **205** | AGL86 | AGL6 |
| **68** | AGL21 | AG | **206** | AGL62 | AGL86 |
| **69** | SVP | AGL6 | **207** | AGL86 | AGL65 |
| **70** | SVP | AP1 | **208** | AGL86 | AP1 |
| **71** | AGL24 | SHP1 | **209** | AGL86 | AGL82 |
| **72** | AGL24 | AGL14 | **210** | AGL86 | AGL90 |
| **73** | AGL24 | AGL16 | **211** | FUL | AGL14 |
| **74** | AGL24 | SOC1 | **212** | FUL | SEP4-II |
| **75** | AGL24 | AGL24 | **213** | FUL | AGL6 |
| **76** | AGL24 | AGL6 | **214** | AGL90 | AGL40 |
| **77** | AGL24 | AP1 | **215** | AGL62 | AGL90 |
| **78** | AGL24 | FUL | **216** | AGL103 | AGL92 |
| **79** | AGL24 | AG | **217** | AGL92 | AGL23 |
| **80** | SEP1 | SHP1 | **218** | AGL92 | ABS-II |
| **81** | SEP1 | STK | **219** | AGL92 | SEP4-II |
| **82** | SEP1 | AGL12 | **220** | AGL92 | AGL6 |
| **83** | SEP1 | AGL16 | **221** | AGL62 | AGL92 |
| **84** | SEP1 | SOC1 | **222** | AGL92 | AP1 |
| **85** | SEP1 | AGL21 | **223** | AGL92 | AGL78 |
| **86** | SEP1 | SVP | **224** | AGL92 | AGL90 |
| **87** | SEP1 | AGL24 | **225** | AGL97 | AGL101 |
| **88** | SEP1 | ABS-I | **226** | AGL103 | AGL97 |
| **89** | ABS-II | SEP1 | **227** | AGL97 | AGL104 |
| **90** | AGL42 | SEP1 | **228** | AGL26 | AGL97 |
| **91** | SEP1 | SHP2 | **229** | AGL97 | FLM |
| **92** | SEP1 | AGL6 | **230** | AGL97 | ABS-II |
| **93** | SEP1 | AP1 | **231** | AGL97 | PHERES1 |
| **94** | SEP1 | AGL71 | **232** | AGL97 | SEP4-II |
| **95** | SEP1 | FUL | **233** | AGL97 | AGL43 |
| **96** | SEP1 | AG | **234** | AGL52 | AGL97 |
| **97** | ABS-I | SEP2 | **235** | AGL97 | AGL53 |
| **98** | AGL36 | AGL28 | **236** | AGL97 | AGL6 |
| **99** | AGL62 | AGL36 | **237** | AGL62 | AGL97 |
| **100** | PHERES1 | AGL28 | **238** | AGL97 | AGL65 |
| **101** | AGL38 | AGL28 | **239** | AGL97 | AP1 |
| **102** | AGL103 | AGL39 | **240** | AGL97 | AGL76 |
| **103** | AGL39 | FLM | **241** | AGL77 | AGL97 |
| **104** | AGL39 | PHERES1 | **242** | AGL97 | AGL78 |
| **105** | AGL39 | AGL49 | **243** | AGL97 | AGL82 |
| **106** | AGL52 | AGL39 | **244** | AGL97 | AGL87 |
| **107** | AGL39 | AGL6 | **245** | AGL97 | AGL89 |
| **108** | AGL39 | AP1 | **246** | AGL97 | AGL90 |
| **109** | AGL77 | AGL39 | **247** | AGL97 | AG |
| **110** | AGL39 | AGL78 | **248** | AGL103 | AGL99 |
| **111** | AGL39 | AGL82 | **249** | AGL99 | PHERES1 |
| **112** | AGL39 | AG | **250** | AGL99 | AGL43 |
| **113** | AP1 | SEP4-II | **251** | AGL52 | AGL99 |
| **114** | AGL40 | PHERES1 | **252** | AGL99 | AGL76 |
| **115** | AGL40 | AGL38 | **253** | AGL77 | AGL99 |
| **116** | AGL42 | AGL16 | **254** | AGL99 | AGL78 |
| **117** | AGL42 | AGL6 | **255** | AGL99 | AGL82 |
| **118** | AGL45 | AGL40 | **256** | SEP3 | SHP1 |
| **119** | AGL49 | AGL74-II | **257** | SEP3 | STK |
| **120** | SEP2 | AGL16 | **258** | SEP3 | AGL16 |
| **121** | AGL52 | AGL55 | **259** | SEP3 | SOC1 |
| **122** | AGL52 | AGL74-II | **260** | SEP3 | SVP |
| **123** | AGL53 | AGL16 | **261** | SEP3 | AGL24 |
| **124** | AGL53 | AGL6 | **262** | SEP3 | ABS-I |
| **125** | AGL54 | AGL83 | **263** | ABS-II | SEP3 |
| **126** | AGL52 | AGL56 | **264** | SEP3 | SHP2 |
| **127** | SHP2 | AGL6 | **265** | SEP3 | AGL6 |
| **128** | AGL62 | PHERES1 | **266** | SEP3 | AP1 |
| **129** | AGL62 | AGL38 | **267** | SEP3 | FUL |
| **130** | AGL62 | AGL90 | **268** | SEP3 | AG |
| **131** | AGL64 | AGL48 | **269** | AG | AGL13 |
| **132** | AGL77 | AGL64 | **270** | AG | AGL16 |
| **133** | AGL66 | SEP4-II | **271** | AG | SEP2 |
| **134** | AGL66 | AGL6 | **272** | AG | FUL |
| **135** | AGL66 | AGL65 | **273** | PI | AP3 |
| **136** | AGL66 | AGL82 | **274** | AP3 | PI |
| **137** | AGL66 | AGL90 |  |  |  |
| **138** | AGL66 | AGL96 |  |  |  |

**Table S3: Ternary complexes identified in the large-scale matrix-based yeast three-hybrid screening.**

The classification in the 5^th^ column is made based on Pařenicová et al [5].. The δ subgroup is also known as the MIKC* class of MADS box proteins [6]. In the last column the tissue is indicated in which the genes encoding the interacting proteins are showing the strongest co-expression (Based on the AtGenExpress data for wild type plants [7]). A question mark indicates that one of the genes encoding the interacting proteins is not present on the array. The numbers refer to the following descriptions:

ATGE_8 = shoot apex, transition (before bolting);

ATGE_26 = cauline leaves;

ATGE_27 = stem, 2nd internode;

ATGE_28 = 1st node;

ATGE_43 = flowers stage 15, stamen;

ATGE_45 = flowers stage 15, carpels;

ATGE_73 = mature pollen;

ATGE_83 = seeds, stage 9, w/o siliques; curled cotyledons to early green cotyledons

Embryos;

ATGE_84 = seeds, stage 10, w/o siliques; green cotyledons embryos;

ATGE_92 = flower;

ATGE_99 = root.

| ***Complex number*** | ***pADGAL4***  ***insert*** | ***pTFT1***  ***insert*** | ***pBDGAL4***  ***insert*** | ***Classification*** | ***Co-express.*** |
| --- | --- | --- | --- | --- | --- |
| **1** | AGL103 | AGL102 | AGL101 | β-α-β | ? |
| **2** | AGL103 | AGL55 | AGL101 | β-α-β | ? |
| **3** | AGL103 | AGL56 | AGL101 | β-α-β | ? |
| **4** | AGL104 | AGL65 | AGL104 | δ-δ-δ | ATGE_73 |
| **5** | SOC1 | AGL19 | AGL17 | MIKC-MIKC-MIKC | ATGE_99 |
| **6** | SOC1 | AGL6 | AG | MIKC-MIKC-MIKC | ATGE_92 |
| **7** | AGL21 | SOC1 | AP1 | MIKC-MIKC-MIKC | ATGE_84 |
| **8** | AGL21 | SOC1 | FUL | MIKC-MIKC-MIKC | ATGE_73 |
| **9** | AGL24 | SOC1 | SEP4-II | MIKC-MIKC-MIKC | ATGE_27 |
| **10** | AGL24 | SOC1 | SEP2 | MIKC-MIKC-MIKC | ATGE_8 |
| **11** | AGL24 | SOC1 | AGL17 | MIKC-MIKC-MIKC | ATGE_73 |
| **12** | AGL24 | SOC1 | AGL42 | MIKC-MIKC-MIKC | ATGE_28 |
| **13** | AGL24 | FUL | SEP4-II | MIKC-MIKC-MIKC | ATGE_26 |
| **14** | SEP1 | SHP1 | AGL14 | MIKC-MIKC-MIKC | ATGE_73 |
| **15** | SEP1 | SOC1 | SEP4-II | MIKC-MIKC-MIKC | ATGE_26 |
| **16** | SEP1 | SOC1 | SEP2 | MIKC-MIKC-MIKC | ATGE_73 |
| **17** | SEP1 | SOC1 | AGL14 | MIKC-MIKC-MIKC | ATGE_73 |
| **18** | SEP1 | ABS-I | SEP2 | MIKC-MIKC-MIKC | ATGE_45 |
| **19** | SEP1 | ABS-II | AGL14 | MIKC-MIKC-MIKC | ATGE_73 |
| **20** | SEP1 | ABS-II | AGL63 | MIKC-MIKC-MIKC | ? |
| **21** | SEP1 | SHP2 | AGL14 | MIKC-MIKC-MIKC | ATGE_84 |
| **22** | AGL39 | AGL103 | AGL55 | α-β-α | ? |
| **23** | SEP4-II | AP1 | AGL24 | MIKC-MIKC-MIKC | ATGE_8 |
| **24** | AGL66 | AGL65 | AGL104 | δ-δ-δ | ATGE_73 |
| **25** | AGL73 | AGL26 | AGL80 | α-β-γ | ? |
| **26** | AGL74II | AGL103 | AGL101 | α-β-β | ? |
| **27** | AGL74II | AGL103 | AGL102 | α-β-α | ? |
| **28** | AGL74N | ABS-I | SEP2 | α-MIKC-MIKC | ? |
| **29** | AGL74N | AGL65 | AGL104 | α-δ-δ | ? |
| **30** | AGL78 | AGL74II | AGL79 | β-α-MIKC | ? |
| **31** | AGL78 | AGL74II | AGL101 | β-α-β | ? |
| **32** | AGL101 | AGL84 | AGL79 | β-α-MIKC | ATGE_73 |
| **33** | AGL52 | AGL84 | AGL74II | β-α-α | ? |
| **34** | AGL86 | AGL6 | AG | γ-MIKC-MIKC | ATGE_83 |
| **35** | FUL | SEP4-II | SVP | MIKC-MIKC-MIKC | ATGE_26 |
| **36** | AGL92 | AGL103 | AGL40 | γ-β-α | ATGE_73 |
| **37** | AGL92 | AGL103 | AGL55 | γ-β-α | ? |
| **38** | AGL92 | AGL103 | AGL56 | γ-β-α | ? |
| **39** | AGL92 | ABS-II | SEP2 | γ-MIKC-MIKC | ATGE_73 |
| **40** | AGL62 | AGL92 | AGL40 | α-γ-α | ATGE_73 |
| **41** | AGL92 | AGL78 | AGL55 | γ-β-α | ? |
| **42** | AGL103 | AGL99 | AGL101 | β-α-β | ATGE_73 |
| **43** | AGL99 | AGL78 | ABS-II | α-β-MIKC | ? |
| **44** | SEP3 | SHP1 | AGL14 | MIKC-MIKC-MIKC | ATGE_83 |
| **45** | SEP3 | SOC1 | SEP2 | MIKC-MIKC-MIKC | ATGE_43 |
| **46** | SEP3 | SOC1 | AGL14 | MIKC-MIKC-MIKC | ATGE_83 |
| **47** | SEP3 | AG | AGL14 | MIKC-MIKC-MIKC | ATGE_83 |

**Table S4: Additional dimers identified in the large-scale yeast three-hybrid screening.**

| **Identified dimers** |
| --- |
| SOC1-AGL98 |
| AGL74N-AGL79 |
| AGL74N-AGL102 |
| AGL97-AGL38 |
| AGL97- AGL79 |
| AGL99-AGL38 |
| AGL99- AGL79 |
| AGL102-AGL97 |
| AGL102-AGL99 |

**Table S5: Prediction of activation domains in MADS box transcription factors.**

Results of DILIMOT analysis of auto-activating MADS box sequences. Motifs were found using DILIMOT as described in Material and Methods. Each motif contains both unambiguous positions (capital letter for each amino acid) and ambiguous positions (x). N_a_, number of auto-activating sequences containing the motif; N_n_, number of non-auto-activating sequences containing the motif; f_a_, percentage of auto-activating sequences containing the motif; f_n_, percentage of non-auto-activating sequences containing the motif. TF_gen, percentage of Arabidopsis proteins containing the motif that are transcription factors. For all 10 motifs, this percentage is higher than the percentage of all Arabidopsis proteins that are transcription factors (5.7%): from all Arabidopsis proteins with a motif match, on average 13% is classified as transcription factor, whereas transcription factors constitute only 5,7% of all Arabidopsis proteins. This overrepresentation is even much stronger when requiring two motifs to be present: 24% of all proteins containing two of the motifs are transcription factors.

‘Selected’ indicates whether the motif was used by a decision tree model that was built to predict auto-activation based on occurrence of the motifs; those that were selected presumably are the most relevant motifs. Two of these selected motifs overlap with the previously identified motifs for AP1 (HQxQ overlaps the glutamine rich domain and NxxxxxQE overlaps the acidic domain) [8]. Arabidopsis transcription factors were obtained via the AGRIS website [9]

| **motif** | **N_a_** | **N_n_** | **f_a_ (%)** | **f_n_(%)** | **TF_gen(%)** | **Selected** |
| --- | --- | --- | --- | --- | --- | --- |
| MGxxxxxN | 5 | 1 | 26 | 2 | 9.0 | Y |
| NxNxxxF | 7 | 4 | 37 | 6 | 12.6 | N |
| QxxxFxxN | 6 | 4 | 32 | 6 | 9.0 | N |
| QxQH | 6 | 8 | 32 | 12 | 17.3 | N |
| QxQxxN | 7 | 12 | 37 | 18 | 14.3 | Y |
| HQxQ | 8 | 6 | 42 | 9 | 18.5 | Y |
| NxNQ | 6 | 8 | 32 | 12 | 13.7 | N |
| NxxxxxQE | 7 | 6 | 37 | 9 | 9.1 | Y |
| QxQxxxxN | 6 | 8 | 32 | 12 | 14.8 | N |
| NxNxxxxS | 9 | 13 | 47 | 20 | 13.1 | Y |

**Table S6: The set of SEP3ΔC2 dimers that has been generated in yeast strain PJ69-4A.**

Number 288 (PI-SEP3 ΔC2) was never found as dimer, but has been generated as negative control.

| ***Dimer number*** | ***pADGAL4 insert*** | ***pTFT1 insert*** |
| --- | --- | --- |
| **275** | AG | SEP3ΔC2 |
| **276** | SHP1 | SEP3ΔC2 |
| **277** | SHP2 | SEP3ΔC2 |
| **278** | AGL6 | SEP3ΔC2 |
| **279** | AP1 | SEP3ΔC2 |
| **280** | FUL | SEP3ΔC2 |
| **281** | STK | SEP3ΔC2 |
| **282** | AGL16 | SEP3ΔC2 |
| **283** | SOC1 | SEP3ΔC2 |
| **284** | SVP | SEP3ΔC2 |
| **285** | AGL24 | SEP3ΔC2 |
| **286** | ABS-I | SEP3ΔC2 |
| **287** | ABS-II | SEP3ΔC2 |
| **288** | PI | SEP3ΔC2 |
| **289** | SEP1 | SEP3ΔC2 |

**Table S7: Ternary complexes identified in the matrix-based yeast three-hybrid screening for SEP3ΔC2.**

The classification in the 5^th^ column is made based on Pařenicová et al [5]. In the last column the tissue is indicated in which the genes encoding the interacting proteins are showing the strongest co-expression (Based on the AtGenExpress data for wild type plants [7]). A question mark indicates that at least one of the genes encoding the interacting proteins is not present on the ATH1 array. Note that for these combinations it is possible that the proteins are not co-expressed and hence that the in yeast identified complexes are not biological relevant and represent false positives. The numbers refer to the following descriptions:

ATGE_8 = shoot apex, transition (before bolting);

ATGE_29 = shoot apex, inflorescence (after bolting);

ATGE_31 = flowers stage 9;

ATGE_33 = flowers stage 12;

ATGE_35 = flowers stage 12, petals;

ATGE_36 = flowers stage 12, stamens;

ATGE_37 = flowers stage 12, carpels;

ATGE_45 = flowers stage 15, carpels;

ATGE_73 = mature pollen;

ATGE_76 = siliques, w/ seeds stage 3; mid globular to early heart embryos;

ATGE_78 = siliques, w/ seeds stage 5; late heart to mid torpedo embryos;

ATGE_81 = seeds, stage 7, w/o siliques; late torpedo to early walking-stick embryos;

ATGE_83 = seeds, stage 9, w/o siliques; curled cotyledons to early green cotyledons

Embryos;

| ***Complex number*** | ***pADGAL4***  ***insert*** | ***pTFT1***  ***insert*** | ***pBDGAL4***  ***insert*** | ***Classification*** | ***Co-Express.*** |
| --- | --- | --- | --- | --- | --- |
| **48** | AP3 | PI | SEP3ΔC2 | MIKC-MIKC-MIKC | ATGE_35 |
| **49** | AGL15 | STK | SEP3ΔC2 | MIKC-MIKC-MIKC | ATGE_81 |
| **50** | AGL15 | AP1 | SEP3ΔC2 | MIKC-MIKC-MIKC | ATGE_78 |
| **51** | SEP1 | SHP1 | SEP3ΔC2 | MIKC-MIKC-MIKC | ATGE_37 |
| **52** | SEP1 | AGL24 | SEP3ΔC2 | MIKC-MIKC-MIKC | ATGE_8 |
| **53** | SEP1 | ABS-I | SEP3ΔC2 | MIKC-MIKC-MIKC | ATGE_45 |
| **54** | ABS-II | SEP1 | SEP3ΔC2 | MIKC-MIKC-MIKC | ATGE_45 |
| **55** | SEP1 | SHP2 | SEP3ΔC2 | MIKC-MIKC-MIKC | ATGE_37 |
| **56** | SEP1 | AP1 | SEP3ΔC2 | MIKC-MIKC-MIKC | ATGE_31 |
| **57** | AGL92 | ABS-II | SEP3ΔC2 | γ-MIKC-MIKC | ATGE_83 |
| **58** | SEP3 | SHP1 | SEP3ΔC2 | MIKC-MIKC-MIKC | ATGE_37 |
|  | SHP1 | SEP3ΔC2 | SHP1 | MIKC-MIKC-MIKC | ATGE_37 |
| **59** | SEP3 | SOC1 | SEP3ΔC2 | MIKC-MIKC-MIKC | ATGE_29 |
| **60** | SEP3 | ABS-I | SEP3ΔC2 | MIKC-MIKC-MIKC | ATGE_45 |
| **61** | SEP3 | ABS-II | SEP3ΔC2 | MIKC-MIKC-MIKC | ATGE_45 |
| **62** | AG | SEP3ΔC2 | AG | MIKC-MIKC-MIKC | ATGE_36 |
|  | SEP3 | AG | SEP3ΔC2 | MIKC-MIKC-MIKC | ATGE_36 |
| **63** | AG | SEP3ΔC2 | SHP1 | MIKC-MIKC-MIKC | ATGE_37 |
|  | SHP1 | SEP3ΔC2 | AG | MIKC-MIKC-MIKC | ATGE_37 |
| **64** | AG | SEP3ΔC2 | SHP2 | MIKC-MIKC-MIKC | ATGE_37 |
|  | SHP2 | SEP3ΔC2 | AG | MIKC-MIKC-MIKC | ATGE_37 |
| **65** | AG | SEP3ΔC2 | AP1 | MIKC-MIKC-MIKC | ATGE_29 |
| **66** | AG | SEP3ΔC2 | ABS-I | MIKC-MIKC-MIKC | ATGE_45 |
| **67** | AG | SEP3ΔC2 | ABS-II | MIKC-MIKC-MIKC | ATGE_45 |
|  | ABS-II | SEP3ΔC2 | AG | MIKC-MIKC-MIKC | ATGE_45 |
| **68** | SHP1 | SEP3ΔC2 | AP1 | MIKC-MIKC-MIKC | ATGE_33 |
| **69** | SHP1 | SEP3ΔC2 | SVP | MIKC-MIKC-MIKC | ATGE_83 |
| **70** | SHP1 | SEP3ΔC2 | ABS-I | MIKC-MIKC-MIKC | ATGE_45 |
| **71** | SHP1 | SEP3ΔC2 | ABS-II | MIKC-MIKC-MIKC | ATGE_45 |
|  | ABS-II | SEP3ΔC2 | SHP1 | MIKC-MIKC-MIKC | ATGE_45 |
| **72** | SHP1 | SEP3ΔC2 | ANR1 | MIKC-MIKC-MIKC | ATGE_81 |
| **73** | SHP2 | SEP3ΔC2 | SHP1 | MIKC-MIKC-MIKC | ATGE_37 |
| **74** | SHP2 | SEP3ΔC2 | SHP2 | MIKC-MIKC-MIKC | ATGE_37 |
| **75** | SHP2 | SEP3ΔC2 | AP1 | MIKC-MIKC-MIKC | ATGE_76 |
| **76** | SHP2 | SEP3ΔC2 | SVP | MIKC-MIKC-MIKC | ATGE_29 |
| **77** | SHP2 | SEP3ΔC2 | AGL24 | MIKC-MIKC-MIKC | ATGE_29 |
|  | AGL24 | SEP3ΔC2 | SHP2 | MIKC-MIKC-MIKC | ATGE_29 |
| **78** | SHP2 | SEP3ΔC2 | ABS-I | MIKC-MIKC-MIKC | ATGE_45 |
| **79** | SHP2 | SEP3ΔC2 | ABS-II | MIKC-MIKC-MIKC | ATGE_45 |
|  | ABS-II | SEP3ΔC2 | SHP2 | MIKC-MIKC-MIKC | ATGE_45 |
| **80** | FUL | SEP3ΔC2 | ABS-I | MIKC-MIKC-MIKC | ATGE_45 |
| **81** | STK | SEP3ΔC2 | AG | MIKC-MIKC-MIKC | ATGE_37 |
| **82** | STK | SEP3ΔC2 | SHP1 | MIKC-MIKC-MIKC | ATGE_37 |
| **83** | STK | SEP3ΔC2 | SHP2 | MIKC-MIKC-MIKC | ATGE_37 |
| **84** | STK | SEP3ΔC2 | AP1 | MIKC-MIKC-MIKC | ATGE_33 |
| **85** | STK | SEP3ΔC2 | FUL | MIKC-MIKC-MIKC | ATGE_37 |
| **86** | STK | SEP3ΔC2 | STK | MIKC-MIKC-MIKC | ATGE_37 |
| **87** | STK | SEP3ΔC2 | SOC1 | MIKC-MIKC-MIKC | ATGE_92 |
| **88** | STK | SEP3ΔC2 | AGL21 | MIKC-MIKC-MIKC | ATGE_73 |
| **89** | STK | SEP3ΔC2 | SVP | MIKC-MIKC-MIKC | ATGE_83 |
| **90** | STK | SEP3ΔC2 | AGL24 | MIKC-MIKC-MIKC | ATGE_83 |
|  | AGL24 | SEP3ΔC2 | STK | MIKC-MIKC-MIKC | ATGE_83 |
| **91** | STK | SEP3ΔC2 | ABS-I | MIKC-MIKC-MIKC | ATGE_45 |
| **92** | STK | SEP3ΔC2 | ABS-II | MIKC-MIKC-MIKC | ATGE_45 |
|  | ABS-II | SEP3ΔC2 | STK | MIKC-MIKC-MIKC | ATGE_45 |
| **93** | AGL16 | SEP3ΔC2 | ABS-I | MIKC-MIKC-MIKC | ATGE_45 |
| **94** | SOC1 | SEP3ΔC2 | AG | MIKC-MIKC-MIKC | ATGE_29 |
| **95** | SOC1 | SEP3ΔC2 | ABS-I | MIKC-MIKC-MIKC | ATGE_77 |
| **96** | SOC1 | SEP3ΔC2 | ABS-II | MIKC-MIKC-MIKC | ATGE_77 |
| **97** | SVP | SEP3ΔC2 | ABS-I | MIKC-MIKC-MIKC | ATGE_73 |
| **98** | AGL24 | SEP3ΔC2 | ABS-I | MIKC-MIKC-MIKC | ATGE_84 |
| **99** | AGL24 | SEP3ΔC2 | ABS-II | MIKC-MIKC-MIKC | ATGE_84 |
| **100** | ABS-II | SEP3ΔC2 | AP1 | MIKC-MIKC-MIKC | ATGE_76 |
| **101** | ABS-II | SEP3ΔC2 | FUL | MIKC-MIKC-MIKC | ATGE_45 |
| **102** | ABS-II | SEP3ΔC2 | AGL14 | MIKC-MIKC-MIKC | ATGE_73 |
| **103** | ABS-II | SEP3ΔC2 | SVP | MIKC-MIKC-MIKC | ATGE_73 |
| **104** | ABS-II | SEP3ΔC2 | ABS-II | MIKC-MIKC-MIKC | ATGE_45 |
| **105** | ABS-II | SEP3ΔC2 | ABS-I | MIKC-MIKC-MIKC | ATGE_45 |
| **106** | PI | SEP3ΔC2 | ABS-I | MIKC-MIKC-MIKC | ATGE_76 |

**FigureS1: Comparison of expression patterns of genes that encode higher-order complex forming proteins.**

The data from the AtGenExpress expression atlas [7] are represented such that expression of each gene is normalized across the entire data set. Blue indicates no expression and red high expression, with yellow for low expression levels of the corresponding gene. The most important groups of tissues are indicated at the top and in detail numbered in the bottom. Below an explanatory list is given for the 79 “AtGeneExpress developmental series” tissues that are represented:

1. ATGE_3 development baseline **Wt roots** 7 days continuous light soil
2. ATGE_9 development baseline **Wt roots** 17 days continuous light soil
3. ATGE_93 comparison with CAGE **Wt root** 15 days long day (16/8) 1x MS agar, 1% sucrose
4. ATGE_94 development on MS agar **Wt root** 8 days continuous light 1x MS agar
5. ATGE_95 development on MS agar **Wt root** 8 days continuous light 1x MS agar, 1% sucrose
6. ATGE_98 development on MS agar **Wt root** 21 days continuous light 1x MS agar
7. ATGE_99 development on MS agar **Wt root** 21 days continuous light 1x MS agar, 1% sucrose
8. ATGE_ 27 development baseline **Wt stem**, 2nd internode 21+ days continuous light soil
9. ATGE_28 development baseline **Wt 1st node** 21+ days continuous light soil
10. ATGE_2 development baseline **Wt hypocotyl** 7 days continuous light soil
11. ATGE_87 phase change **Wt vegetative rosette** 7 days short day (10/14) soil
12. ATGE_89 phase change **Wt vegetative rosette** 14 days short day (10/14) soil
13. ATGE_90 phase change **Wt vegetative rosette** 21 days short day (10/14) soil
14. ATGE_7 development baseline **Wt seedling, green parts** 7 days continuous light, soil.
15. ATGE_96 development on MS agar **Wt seedling, green parts** 8 days continuous light 1x MS agar
16. ATGE_97 development on MS agar **Wt seedling**, **green parts** 8 days continuous light 1x MS agar, 1% sucrose
17. ATGE_100 development on MS agar **Wt seedling, green parts** 21 days continuous light 1x MS agar
18. ATGE_101 development on MS agar **Wt seedling, green parts** 21 days continuous light 1x MS agar, 1% sucrose
19. ATGE_91 comparison with CAGE **Wt leaf** 15 days long day (16/8) 1x MS agar, 1% sucrose
20. ATGE_5 development baseline **Wt leaves** 1 + 2 7 days continuous light soil
21. ATGE_20 development baseline **Wt leaf** 7, proximal half 17 days continuous light soil
22. ATGE_21 development baseline **Wt leaf** 7, distal half 17 days continuous light soil
23. ATGE_1 development baseline **Wt cotyledons** 7 days continuous light soil
24. ATGE_12 development baseline **Wt rosette leaf** # 2 17 days continuous light soil
25. ATGE_13 development baseline **Wt rosette leaf** # 4 17 days continuous light soil
26. ATGE_14 development baseline **Wt rosette leaf** # 6 17 days continuous light soil
27. ATGE_15 development baseline **Wt rosette leaf** # 8 17 days continuous light soil
28. ATGE_16 development baseline **Wt rosette leaf** # 10 17 days continuous light soil
29. ATGE_11 development baseline **gl1-T rosette leaf** #4, 1 cm long 10 days continuous light soil
30. ATGE_10 development baseline **Wt rosette leaf** #4, 1 cm long 10 days continuous light soil
31. ATGE_17 development baseline **Wt rosette leaf** # 12 17 days continuous light soil
32. ATGE_18 development baseline **gl1-T rosette leaf** # 12 17 days continuous light soil
33. ATGE_19 development baseline **Wt leaf** 7, petiole 17 days continuous light soil
34. ATGE_22 development baseline Wt developmental drift, entire **rosette after transition to flowering**, but before bolting 21 days continuous light soil
35. ATGE_23 development baseline Wt developmental drift, entire **rosette after transition to flowering**, 22 days continuous light soil
36. ATGE_24 development baseline Wt developmental drift, entire **rosette after transition to flowering**, 23 days continuous light soil
37. ATGE_26 development baseline **Wt cauline leaves** 21+ days continuous light soil
38. ATGE_34 development baseline Wt flowers stage 12, **sepals** 21+ days continuous light soil
39. ATGE_40 development baseline Wt flowers stage 15, **pedicels** 21+ days continuous light soil
40. ATGE_25 development baseline Wt **senescing leaves** 35 days continuous light soil
41. ATGE_41 development baseline Wt flowers stage 15, **sepals** 21+ days continuous light soil
42. ATGE_4 development baseline **Wt shoot apex**, vegetative + young leaves 7 days continuous light soil
43. ATGE_6 development baseline **Wt shoot apex**, vegetative 7 days continuous light soil
44. ATGE_8 development baseline **Wt shoot apex**, transition (before bolting) 14 days continuous light soil
45. ATGE_29 development baseline **Wt shoot apex**, inflorescence (after bolting) 21 days continuous light soil
46. ATGE_46 development baseline **clv3-7shoot apex**, inflorescence (after bolting) 21+ days continuous light soil
47. ATGE_49 development baseline **ap2-6 shoot apex**, inflorescence (after bolting) 21+ days continuous light soil
48. ATGE_48 development baseline **ap1-15shoot apex**, inflorescence (after bolting) 21+ days continuous light soil
49. ATGE_47 development baseline **lfy-12 shoot apex**, inflorescence (after bolting) 21+ days continuous light soil
50. ATGE_52 development baseline **ufo-1shoot apex**, inflorescence (after bolting) 21+ days continuous light soil
51. ATGE_50 development baseline **ap3-6shoot apex**, inflorescence (after bolting) 21+ days continuous light soil
52. ATGE_51 development baseline **ag-12 shoot apex**, inflorescence (after bolting) 21+ days continuous light soil
53. ATGE_31 development baseline **Wt flowers** stage 9 21+ days continuous light soil
54. ATGE_32 development baseline **Wt flowers** stage 10/11 21+ days continuous light soil
55. ATGE_92 comparison with CAGE **Wt flower** 28 days long day (16/8) soil
56. ATGE_33 development baseline **Wt flowers** stage 12 21+ days continuous light soil
57. ATGE_56 development baseline **ap2-6 flower** stage 12; no sepals or petals 21+ days continuous light soil
58. ATGE_55 development baseline **ap1-15 flower** stage 12; sepals replaced by leaf-like organs, petals mostly lacking, 2° flowers 21+ days continuous light soil
59. ATGE_54 development baseline **lfy-12 flower** stage 12; shoot characteristics; most organs leaf-like 21+ days continuous light soil
60. ATGE_59 development baseline **ufo-1 flower** stage 12; filamentous organs in whorls two and three 21+ days continuous light soil
61. ATGE_57 development baseline **ap3-6 flower** stage 12; no petals or stamens 21+ days continuous light soil
62. ATGE_58 development baseline **ag-12 flower** stage 12; no stamens or carpels 21+ days continuous light soil
63. ATGE_53 development baseline **clv3-7 flower** stage 12; multi-carpel gynoeceum; enlarged meristem; increased 21+ days continuous light soil
64. ATGE_37 development baseline Wt flowers stage 12, **carpels** 21+ days continuous light soil
65. ATGE_35 development baseline Wt flowers stage 12, **petals** 21+ days continuous light soil
66. ATGE_36 development baseline Wt flowers stage 12, **stamens** 21+ days continuous light soil
67. ATGE_39 development baseline **Wt flowers** stage 15 21+ days continuous light soil
68. ATGE_42 development baseline Wt flowers stage 15, **petals** 21+ days continuous light soil
69. ATGE_43 development baseline Wt flowers stage 15, **stamen** 21+ days continuous light soil
70. ATGE_73 **pollen** Wt mature pollen 6 wk continuous light soil
71. ATGE_45 development baseline Wt flowers stage 15, **carpels** 21+ days continuous light soil
72. ATGE_76 seed & silique development **Wt siliques**, w/ seeds stage 3; mid globular to early heart embryos 8 wk long day (16/8) soil
73. ATGE_77 seed & silique development **Wt siliques**, w/ seeds stage 4; early to late heart embryos 8 wk long day (16/8) soil
74. ATGE_78 seed & silique development **Wt siliques**, w/ seeds stage 5; late heart to mid torpedo embryos 8 wk long day (16/8) soil
75. ATGE_79 seed & silique development **Wt seeds**, stage 6, w/o siliques; mid to late torpedo embryos 8 wk long day (16/8) soil
76. ATGE_81 seed & silique development **Wt seeds**, stage 7, w/o siliques; late torpedo to early walking-stick embryos 8 wk long day (16/8) soil
77. ATGE_82 seed & silique development **Wt seeds**, stage 8, w/o siliques; walking-stick to early curled cotyledons embryos 8 wk long day (16/8) soil
78. ATGE_83 seed & silique development **Wt seeds**, stage 9, w/o siliques; curled cotyledons to early green cotyledons 8 wk long day (16/8) soil
79. ATGE_84 seed & silique development **Wt seeds**, stage 10, w/o siliques; green cotyledons embryos 8 wk long day (16/8) soil


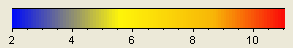

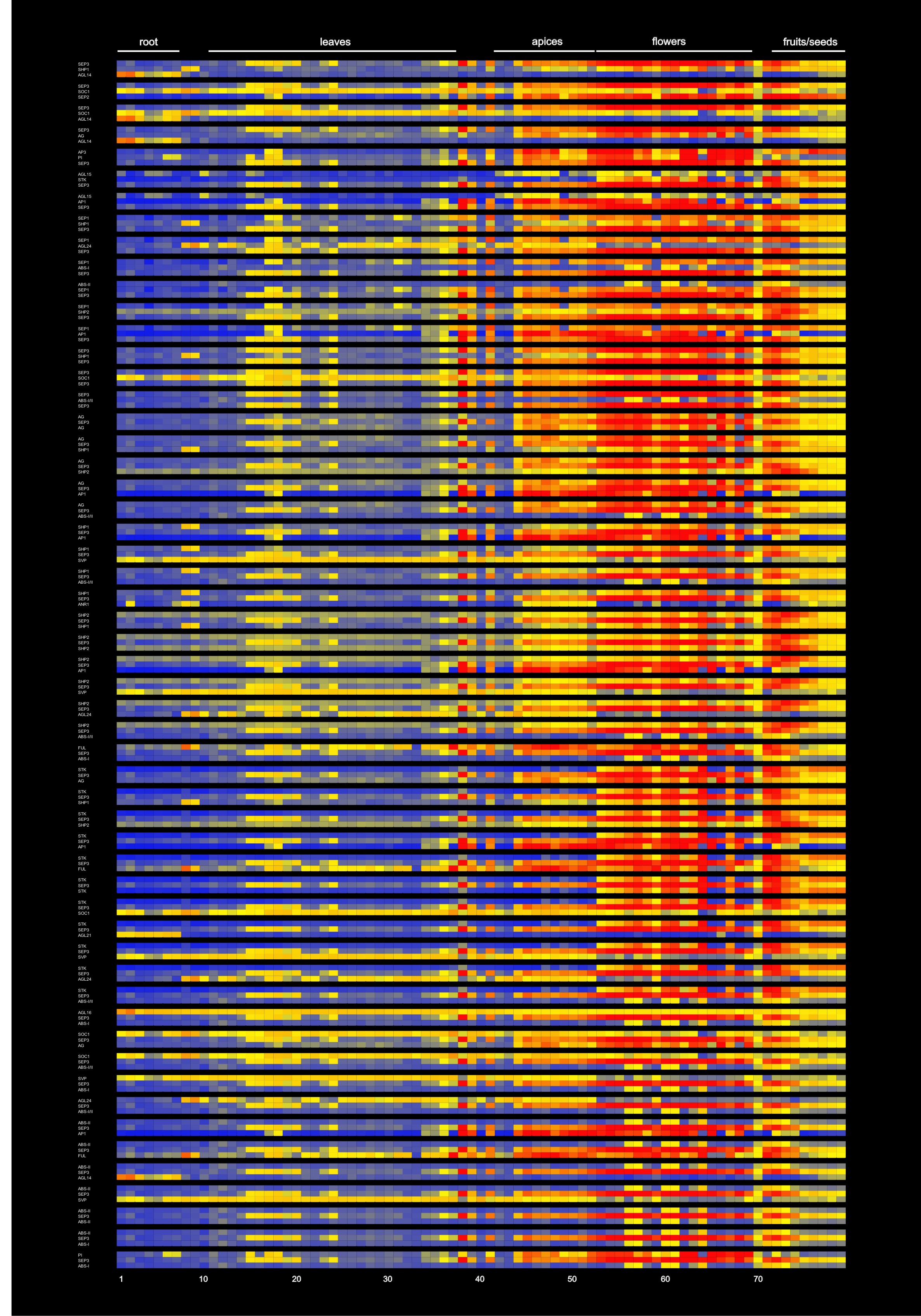


**References**
